# Dynamic regulation of the oxidative stress response by the E3 ligase TRIP12

**DOI:** 10.1016/j.celrep.2025.116262

**Published:** 2025-09-09

**Authors:** Andrew J. Ingersoll, Devlon M. McCloud, Jenny Y. Hu, Michael Rape

**Affiliations:** 1 Department of Molecular and Cell Biology, University of California at Berkeley, Berkeley, CA 94720, USA; 2 Howard Hughes Medical Institute, University of California at Berkeley, Berkeley, CA 94720, USA; 3 California Institute for Quantitative Biosciences (QB3), University of California at Berkeley, Berkeley, CA 94720, USA; 4 Present address: Triana Biomedicines, Lexington, MA 02421, USA; 5 Lead contact

## Abstract

Centered on the transcription factor NRF2 and its E3 ligase CUL3^KEAP1^, the oxidative stress response protects cells from damage by reactive oxygen species (ROS). Increasing ROS inhibits CUL3^KEAP1^ to stabilize NRF2 and elicit antioxidant gene expression, while cells recovering from stress rapidly turn over NRF2 again to prevent reductive stress and oxeiptosis-dependent death. How cells reinitiate NRF2 degradation after ROS have been cleared remains poorly understood. Here, we identify the essential E3 ligase TRIP12 as a crucial component of the oxidative stress response. TRIP12 is a ubiquitin chain elongation factor that cooperates with CUL3^KEAP1^ to ensure robust NRF2 degradation. In this manner, TRIP12 accelerates stress response silencing as oxidative stress is being resolved but limits NRF2 activation during stress. The need for dynamic control of NRF2 degradation therefore comes at the cost of diminished stress signaling, suggesting that TRIP12 inhibition could be used to treat degenerative pathologies characterized by ROS accumulation.

## In brief

The study by Ingersoll et al. identifies the essential E3 ligase TRIP12 as a crucial component of the oxidative stress response. TRIP12 acts as a ubiquitin chain elongation factor that ensures robust NRF2 degradation and stress response silencing when redox homeostasis has been restored.

## Graphical Abstract

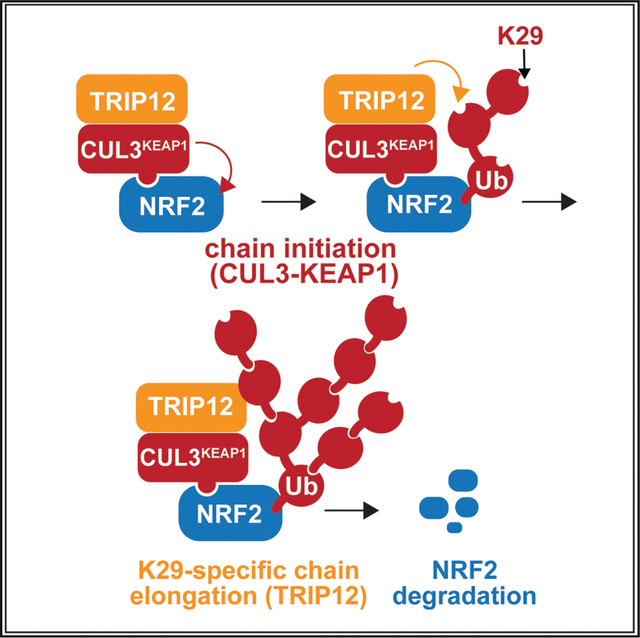

## INTRODUCTION

Reactive oxygen species (ROS), such as superoxide anions, hydroxyl radicals, or hydrogen peroxide, disrupt cell and tissue integrity and thereby accelerate aging and neurodegeneration.^1–3^ Such ROS can result from an exposure to toxins or heavy metals, but also accumulate during infection or due to mutations in genes encoding mitochondrial proteins or ROS-detoxifying enzymes.^1,2^ The cytotoxic effects of ROS are exploited in the clinic during radiation and chemotherapy to eliminate tumor cells and provide patients with cancer with therapeutic benefit.^4^

To counteract deleterious ROS, cells rely on the oxidative stress response, which is centered on the transcription factor NRF2 and its E3 ligase CUL3^KEAP1^.^5–7^ In unstressed cells, CUL3^KEAP1^ ubiquitylates NRF2 to induce its proteasomal degradation and keep NRF2 levels low.^8,9^ As ROS accumulate, oxidation of Cys residues in the substrate adaptor KEAP1 prevents NRF2 turnover, which allows the transcription factor to enter the nucleus and drive antioxidant gene expression.^5,10–13^ While rapid NRF2 stabilization protects cells from ROS, the reciprocal degradation of NRF2 during recovery is also important: a failure to restore NRF2 turnover upon ROS clearance triggers reductive stress, which impairs differentiation and causes a range of developmental and metabolic diseases.^14–19^ Persistent inhibition of KEAP1 also induces cell death through oxeiptosis.^20^ In line with these observations, *KEAP1* deletion in mice causes perinatal lethality,^21^ and mutations in *KEAP1* are drivers of lung adenocarcinoma and other tumors.^22,23^ Cells must therefore balance their need to stabilize NRF2 during stress with an ability to quickly turn over NRF2 once conditions improve. How cells establish such dynamic control of oxidative stress signaling is not fully understood.

CUL3^KEAP1^ differs from other ubiquitylation enzymes that elicit proteasomal degradation. While most E3 ligases bind their substrates very briefly,^24^ KEAP1 can stably interact with NRF2 and sequester it in the cytoplasm.^25,26^ Although ROS prevent NRF2 turnover, they do not impede the ability of KEAP1 to bind the transcription factor,^10,27,28^ and KEAP1 variants known as “anchor mutants” even show increased affinity for NRF2 without triggering degradation.^29^ Moreover, heterobifunctional small molecules that recruit CUL3^KEAP1^ to proteins often do not elicit turnover.^30^ These results mirror other CUL3 E3 ligases that rely on distinct substrate adaptors but use the same catalytic module to monoubiquitylate, but not degrade, proteins.^31–34^ These findings indicate that CUL3^KEAP1^ may not be sufficient to induce robust NRF2 degradation, but whether other ubiquitylation enzymes are required is not known.

Aside from NRF2, CUL3^KEAP1^ targets the PALB2 protein, which recruits BRCA2 to sites of DNA damage.^35^ Ubiquitylation does not change the abundance of PALB2, but prevents its binding to BRCA2 to avoid homologous recombination repair in the G1 phase of the cell cycle. KEAP1 also associates with the phosphatase PGAM5,^36^ an interaction that is regulated by ROS during oxeiptosis.^20^ In addition, CUL3^KEAP1^ engages p62 (SQSTM1), which controls early steps in autophagy.^37,38^ Rather than inducing degradation, CUL3^KEAP1^ ubiquitylates p62 to release an autoinhibitory conformation for recognition of autophagic cargo.^39^ p62 engages KEAP1 so stably that it is sequestered from other substrates,^40–42^ which impedes NRF2 turnover and thereby elicits p62 expression.^37,43^ These observations highlight that CUL3^KEAP1^ often does not induce proteasomal turnover. How CUL3^KEAP1^ can trigger efficient NRF2 degradation therefore requires further investigation.

Here, we identify the essential E3 ligase TRIP12, which has been reported to control cell proliferation and is dysregulated in neurological diseases^44–50^ as a component of the oxidative stress response. TRIP12 is a ubiquitin chain elongation factor that cooperates with CUL3^KEAP1^ to decorate NRF2 with K29-linked conjugates known to drive proteasomal degradation. Chain extension by TRIP12 accelerates stress response silencing upon ROS clearance but restricts NRF2 function during stress. Efficient degradation of NRF2 through CUL3^KEAP1^ therefore requires an additional chain elongation factor, TRIP12, whose inhibition could be exploited to prolong antioxidant signaling in degenerative pathologies driven by ROS accumulation.

## RESULTS

### CCNF is required for oxidative stress signaling in myoblasts

To determine how cells ensure dynamic regulation of the oxidative stress response, we turned to myoblasts, which require NRF2 degradation for successful differentiation. As seen before,^14,15,34^ depletion of KEAP1 and the concomitant stabilization of NRF2 interfered with myotube formation (Figure S1A). Lowering NRF2 levels overcame the differentiation block in the absence of KEAP1 (Figure S1A).^15^ Thus, depleting any regulators of the oxidative stress response that ensure NRF2 function should restore myotube formation despite low levels of KEAP1.

**Figure 1.**
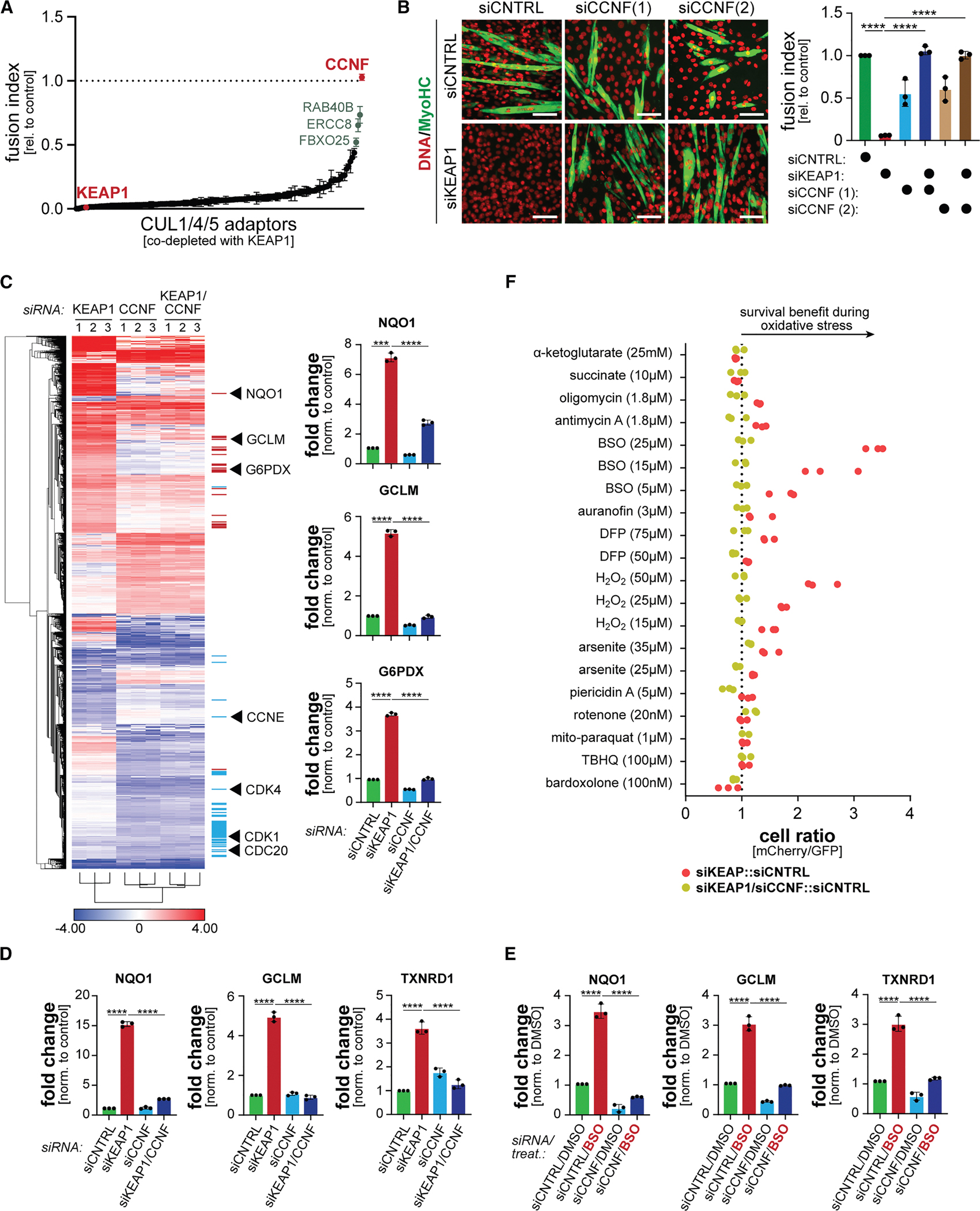
CCNF is required for oxidative stress signaling in myoblasts (A) A focused genetic screen identifies CCNF as a candidate regulator of oxidative stress signaling in myoblasts. C2C12 myoblasts were depleted of substrate adaptors or CUL1, CUL4, and CUL5, as well as KEAP1. Myoblasts were induced to differentiate, and myotube formation was followed by immunostaining against myosin heavy chain (MyHC). *n* = 2 independent screens. (B) C2C12 myoblasts were depleted of either KEAP1 or CCNF (using independent siRNAs) or both, induced to differentiate, and analyzed by microscopy against MyHC. Left: microscopy pictures; scale bars, 100 μm. Right: quantification of three independent experiments. Data are represented as the mean ± standard deviation. ****p < 0.0001. (C) RNA-sequencing analysis of C2C12 myoblasts that were depleted of either KEAP1 or CCNF or both. Red lines, NRF2 target genes; blue lines, E2F target genes. Right: quantification of gene expression for select NRF2 targets. Data are represented as the mean ± standard deviation. ***p < 0.001 and ****p < 0.0001. (D) C2C12 myoblasts were depleted of KEAP1, CCNF, or both, and expression of select NRF2 targets was analyzed by qPCR. Data are represented as the mean ± standard deviation. ****p < 0.0001. (E) C2C12 myoblasts were treated with 25 μM BSO, depleted of CCNF, or both, and the expression of select NRF2 targets was analyzed by qPCR. Data are represented as the mean ± standard deviation. ****p < 0.0001. (F) GFP-labeled control cells were mixed at a 1:1 ratio with mCherry-labeled cells depleted of KEAP1 (red dots). CCNF was depleted together with KEAP1 as indicated (yellow dots). Cells were exposed to increasing concentrations of oxidative stressors. After 2 days, the ratio of mCherry- to GFP-labeled cells was determined by flow cytometry. *n* = 3 independent experiments. See also Figure S1 and Table S1.

We had previously used this screening paradigm to probe substrate adaptors of CUL2 and CUL3 E3 ligases and thereby discovered the CUL2^FEM1B^-dependent reductive stress response.^15^ We now interrogated the remaining Cullin-RING E3 ligases (CUL1, CUL4A/B, and CUL5) and asked if depletion of their substrate adaptors enabled myoblast differentiation in the absence of KEAP1. Targeting the CUL1 adaptor CCNF allowed for myotube formation in cells depleted of KEAP1 (Figure 1A; Table S1), although reducing CCNF by itself slightly impaired this cell fate decision (Figure S1B). We validated these results with independent small interfering RNAs (siRNAs), which confirmed that reducing CCNF enabled the differentiation of KEAP1-depleted myoblasts (Figure 1B).

To test if CCNF impacted myogenesis through NRF2, we subjected myoblasts depleted of KEAP1, CCNF, or both to RNA sequencing. As expected, lowering KEAP1 increased the mRNA levels of NRF2 targets (Figure 1C). By contrast, depleting CCNF altered the expression of cell-cycle regulators, likely due to the role of SCF^CCNF^ in degrading E2F transcription factors.^51^ Cells depleted of both KEAP1 and CCNF showed the same altered expression of cell-cycle regulators as caused by lowering CCNF, yet did not induce NRF2 targets (Figure 1C). qPCR analyses confirmed that loss of CCNF blunted the expression of NRF2 targets, even if KEAP1 levels were low and NRF2 should have been stabilized (Figure 1D), an observation we also made when KEAP1 was inhibited by chemical inducers of oxidative stress (Figures 1E and S1C). These results were mirrored by changes in ROS: while KEAP1 depletion reduced ROS dependent on NRF2 (Figure S1D), lowering CCNF had the opposite effect and reequilibrated ROS in the absence of KEAP1 (Figure S1E). The depletion of CCNF therefore not only results in cell-cycle defects^52–54^ but also impedes NRF2 activation and ROS scavenging.

Having seen its effects on NRF2 target gene expression, we asked if CCNF depletion impacted survival during oxidative stress using cell competition.^55^ To establish this approach, we mixed equal numbers of GFP-labeled control cells with mCherry-labeled cells depleted of KEAP1. After adding oxidative stressors, such as the glutathione synthesis inhibitor buthionine sulfoximine (BSO), we measured the ratio of GFP- to mCherry-labeled cells by flow cytometry. As expected, depleting KEAP1 improved myoblast survival dependent on the NRF2 that accumulates under these conditions (Figure S1F). The protective effect of KEAP1 depletion was apparent if cells experienced strong oxidative stress, showing that some CUL3^KEAP1^ remains active despite high ROS, and this CUL3^KEAP1^ compromises the ability of cells to survive such stress. Mirroring its effects on NRF2 target gene expression, depleting CCNF eliminated the fitness benefit provided by KEAP1 inhibition during both moderate and strong oxidative stress (Figure 1F).

We conclude that CCNF is required for oxidative stress signaling in myoblasts. CCNF is best known for targeting cell-cycle regulators for degradation, thereby allowing cells to progress through the S and G2 phases of their cell cycle.^51,52,54,56^ However, mutations in *CCNF* also cause amyotrophic lateral sclerosis (ALS) and frontotemporal dementia (FTD),^57–60^ two diseases that are characterized by ROS accumulation.^61^ It is possible that defective oxidative stress signaling, as described here, contributes to the emergence of ALS or FTD in patients with *CCNF* mutations.

### p62/SQSTM supports oxidative stress signaling

**Figure 2.**
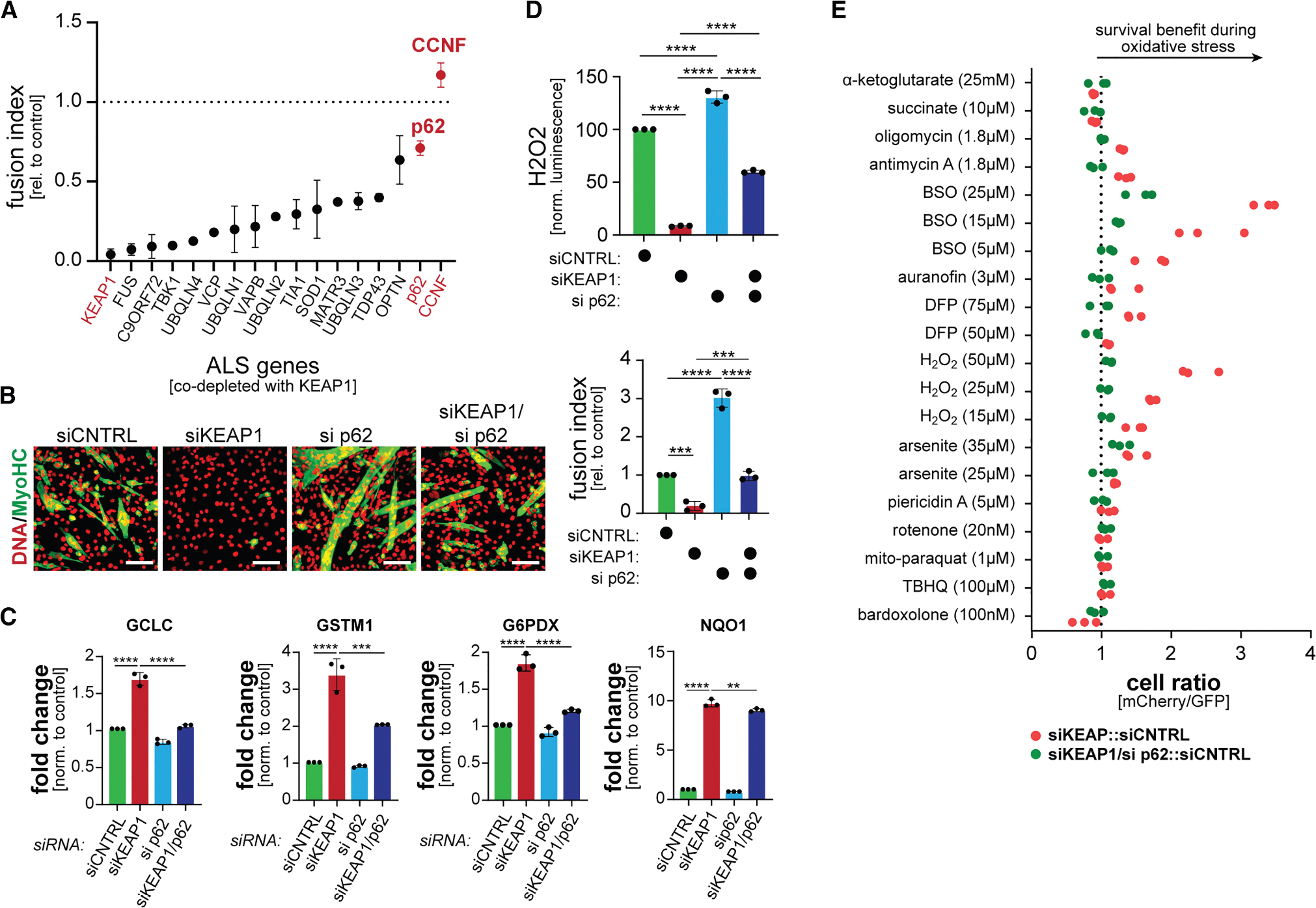
p62 supports oxidative stress signaling in myoblasts (A) C2C12 myoblasts were depleted of KEAP1 and proteins encoding risk factors for amyotrophic lateral sclerosis or frontotemporal dementia and induced to differentiate. Myotube formation was monitored by microscopy against MyHC. *n* = 2 independent screens. (B) C2C12 myoblasts were depleted of KEAP1, p62, or both and induced to differentiate. Myotube formation was monitored by immunofluorescence microscopy against MyHC. Left: microscopy images of differentiation; scale bars, 100 μm. Right: quantification of three independent experiments. (C) C2C12 myoblasts were depleted of KEAP1, p62, or both, and the expression of NRF2 targets was determined by qPCR. *n* = 3 replicates. Data are represented as mean ± standard deviation. **p < 0.01, ***p < 0.001, and ****p < 0.0001. (D) C2C12 myoblasts were depleted of KEAP1, p62, or both, and intracellular ROS levels were determined by a ROS-Glo luciferase assay. Data are represented as the mean ± standard deviation. ****p < 0.0001. (E) GFP-labeled C2C12 myoblasts were mixed with mCherry-labeled cells that had been depleted of KEAP1 (red dots) or both p62 and KEAP1 (green dots). Cells were exposed to increasing concentrations of oxidative stressors. After 2 days, the ratio of GFP- to mCherry-labeled cells was determined by flow cytometry. Three independent experiments are shown (KEAP1-depleted cells are the same as in Figure 1F).

Having seen the effects of CCNF depletion on NRF2 function, we asked if we could use myoblast differentiation to link other ALS risk factors to oxidative stress signaling. We therefore depleted proteins mutated in ALS together with KEAP1 and monitored myotube formation by microscopy. While CCNF depletion showed the strongest phenotype, reducing p62 also allowed for substantial myogenesis despite low KEAP1 (Figure 2A). We confirmed with an independent siRNA that p62 lowering restored myotube formation in the absence of KEAP1 (Figure 2B). Loss of p62 limited the expression of some, but not all, NRF2 targets (Figure 2C), which is in line with its milder differentiation phenotype, and depletion of p62 reversed the reduction in ROS levels that is observed upon NRF2 stabilization (Figure 2D). In line with these results, p62 was required for the survival benefit of KEAP1 depletion during oxidative stress (Figure 2E). As p62 accumulation is known to stabilize NRF2,^62^ these findings underscored that loss of CCNF might also affect, either directly or indirectly, the ability of CUL3^KEAP1^ to mediate NRF2 turnover.

### NRF2 and CUL3^KEAP1^ bind the E3 ligase TRIP12

**Figure 3.**
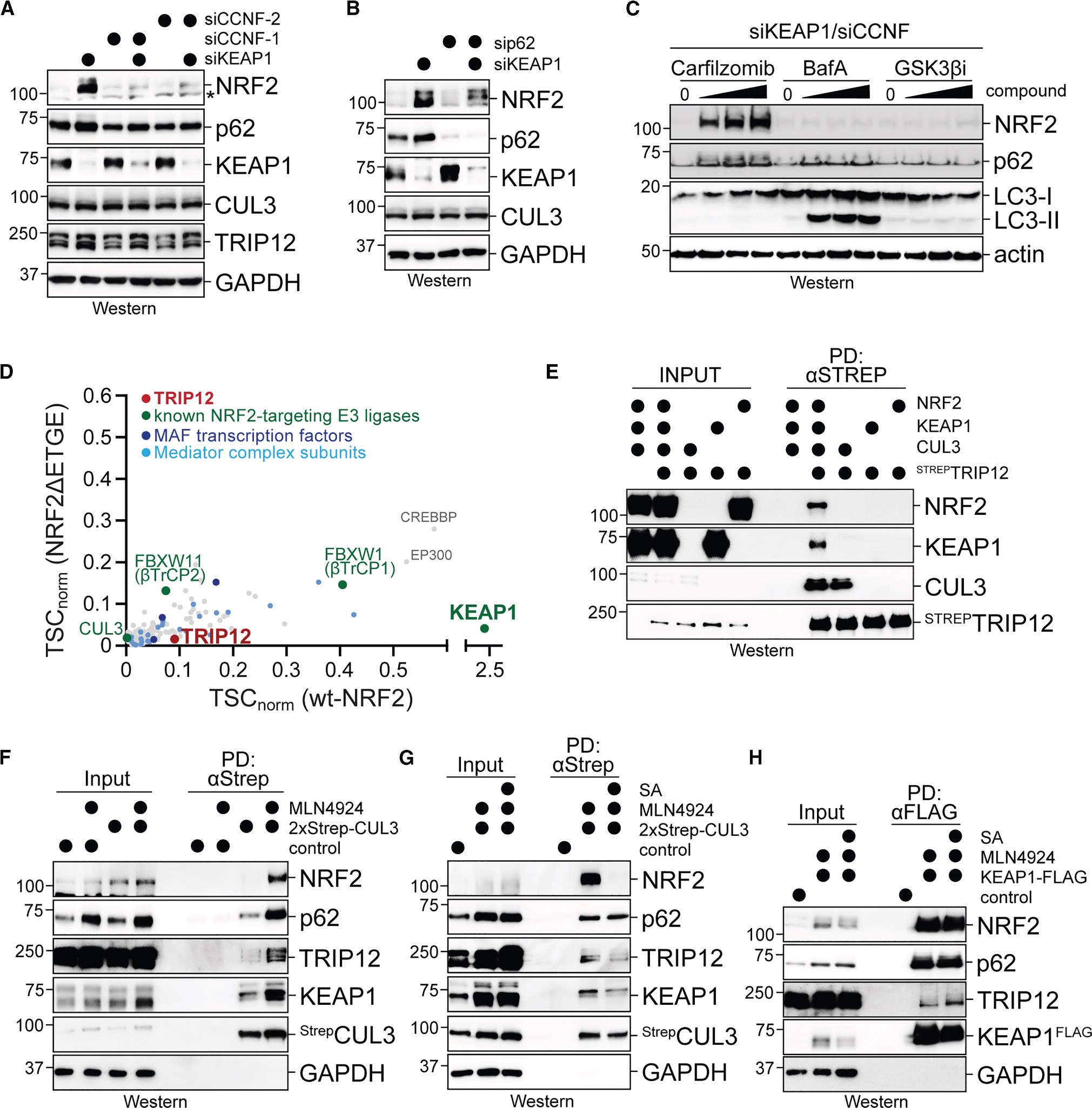
NRF2 and CUL3^KEAP1^ bind TRIP12 (A) C2C12 myoblasts were depleted of KEAP1, CCNF, or both, and NRF2 levels were determined by western blotting. (B) C2C12 myoblasts were depleted of KEAP1, p62, or both, and NRF2 levels were determined by western blotting. (C) C2C12 myoblasts were depleted of KEAP1 and CCNF and treated with increasing concentrations of carfilzomib; bafilomycin A (BafA), or CHIR98014. NRF2 levels were determined by western blotting. (D) ^FLAG^NRF2 or ^FLAG^NRF2^ΔETGE^ was affinity purified from lysates of stably transduced 293T cells that had been treated with MLN4924. Binding partners of NRF2 were determined by mass spectrometry. (E) TRIP12 directly binds CUL3. Immobilized ^STREP^TRIP12 was incubated with CUL3, KEAP1, NRF2, or all proteins at the same time, and bound proteins were detected by western blotting. (F) ^Strep^CUL3 was stably expressed in C2C12 myoblasts, affinity purified, and analyzed for bound proteins by western blotting using specific antibodies. As indicated, MLN4924 was added to delay substrate release from CUL E3 ligases. (G) Stably expressed ^Strep^CUL3 was affinity purified from C2C12 myoblasts, as described in (F). As indicated, sodium arsenite (25 μM; 12 h) was added. MLN4924 was used to prevent substrate degradation. Bound proteins were detected by western blotting. (H) ^FLAG^KEAP1 was transiently expressed in C2C12 myoblasts, affinity purified and analyzed for binding partners by western blotting using specific antibodies. As indicated, sodium arsenite (25 μM; 12 h) was added. See also Figure S2 and Table S2.

To understand how CCNF impacts oxidative stress signaling, we analyzed NRF2 levels in myoblasts depleted of KEAP1, CCNF, or both. While targeting KEAP1 led to the expected increase in NRF2, co-depletion of CCNF had a striking effect and prevented NRF2 accumulation (Figure 3A). Loss of CCNF reduced NRF2 protein levels without impacting NRF2 mRNA abundance (Figures S2A–S2C). p62 depletion also restricted NRF2 levels in cells with low KEAP1 but, consistent with its milder differentiation phenotype, did so less drastically than loss of CCNF (Figure 3B). Treatment of cells co-depleted of CCNF and KEAP1 with the proteasome inhibitor carfilzomib, but not the lysosome inhibitor bafilomycin A, restored NRF2 (Figures 3C and S2D). These findings therefore suggested that loss of CCNF stimulates the proteasomal degradation of NRF2.

As siRNAs only partially deplete their targets, loss of CCNF could either activate any remaining CUL3^KEAP1^ or stimulate a distinct E3 ligase to improve the turnover of NRF2. Previous work had implicated SCF^βTrCP^ in ubiquitylating NRF2,^63^ but inhibition of the kinase GSK3, which is required for SCF^βTrCP^-dependent NRF2 degradation, did not restore NRF2 in the absence of CCNF (Figures 3C and S2D). To identify distinct ubiquitylation enzymes that support NRF2 turnover in the absence of CCNF, we affinity purified NRF2 from cells that were exposed to MLN4924 or that expressed mutant NRF2^ΔETGE^ to inhibit CUL3^KEAP1^-dependent NRF2 degradation. Using mass spectrometry, we found that the two best-known NRF2 E3 ligases, KEAP1 and βTrCP, emerged as abundant NRF2 interactors (Figure 3D; Table S2). E3 ligases that target NRF2 in different cell types, such as HRD1 or WDR23,^64,65^ did not associate with NRF2 in our experiments. In addition, we noted that NRF2 associated with the E3 ligase TRIP12 (Figure 3D; Table S2). Expression of TRIP12 was reported to increase during the S and G2 phases, which accumulate when CCNF is depleted,^50^ suggesting that TRIP12 could, at least in part, account for the improved NRF2 degradation that we observed in cells lacking CCNF.

TRIP12 interacted less efficiently with NRF2^ΔETGE^ and therefore likely engages NRF2 via CUL3^KEAP1^ (Figure 3D). In line with this notion, TRIP12 is correlated with CUL3^KEAP1^ in DepMap to the same extent as the NEDD8-E1 that is required for CUL3 activity (Figure S2E).^66^ Using recombinant proteins, we found that TRIP12 strongly and directly bound CUL3 (Figure 3E), which was independent of the catalytic Cys residue in the HECT domain of TRIP12 (Figure S2F). While TRIP12 did not bind KEAP1 or NRF2 by themselves, it gained access to both proteins through CUL3 (Figure 3E). TRIP12 also bound CUL3 in cells, which was enhanced by MLN4924, which delays substrate release and thereby impedes capture of CUL3 by the inhibitory CAND1 protein (Figure 3F).^67^ If cells experienced oxidative stress, CUL3 lost its association with NRF2 and bound slightly less TRIP12 (Figure 3G). KEAP1 also co-precipitated TRIP12 (Figure 3H), showing that TRIP12 can engage the entire NRF2-E3 ligase, CUL3^KEAP1^, just as we had found with purified proteins.

TRIP12 is an HECT-family E3 ligase that is highly expressed in brain and muscle.^48,49,68^ It can collaborate with other E3 ligases to produce branched ubiquitin chains with abundant K29 linkages, which are known to drive proteasomal degradation.^69–71^ Deletion of *TRIP12* interfered with mouse development,^49^ and its mutation causes Clark-Baraitser syndrome, which is characterized by autism-like symptoms also linked to *CUL3* mutations.^44–46,72,73^ Moreover, TRIP12 is overexpressed in Parkinson’s disease,^47^ a neurodegenerative pathology characterized by ROS accumulation.^61^ Its cell-cycle regulation, its strong binding to CUL3^KEAP1^, and the many parallels between TRIP12 and CUL3^KEAP1^ dysregulation suggested that TRIP12 could support NRF2 degradation to establish the dynamic regulation of the oxidative stress response.

### TRIP12 is a ubiquitin chain elongation factor for CUL3^KEAP1^

**Figure 4.**
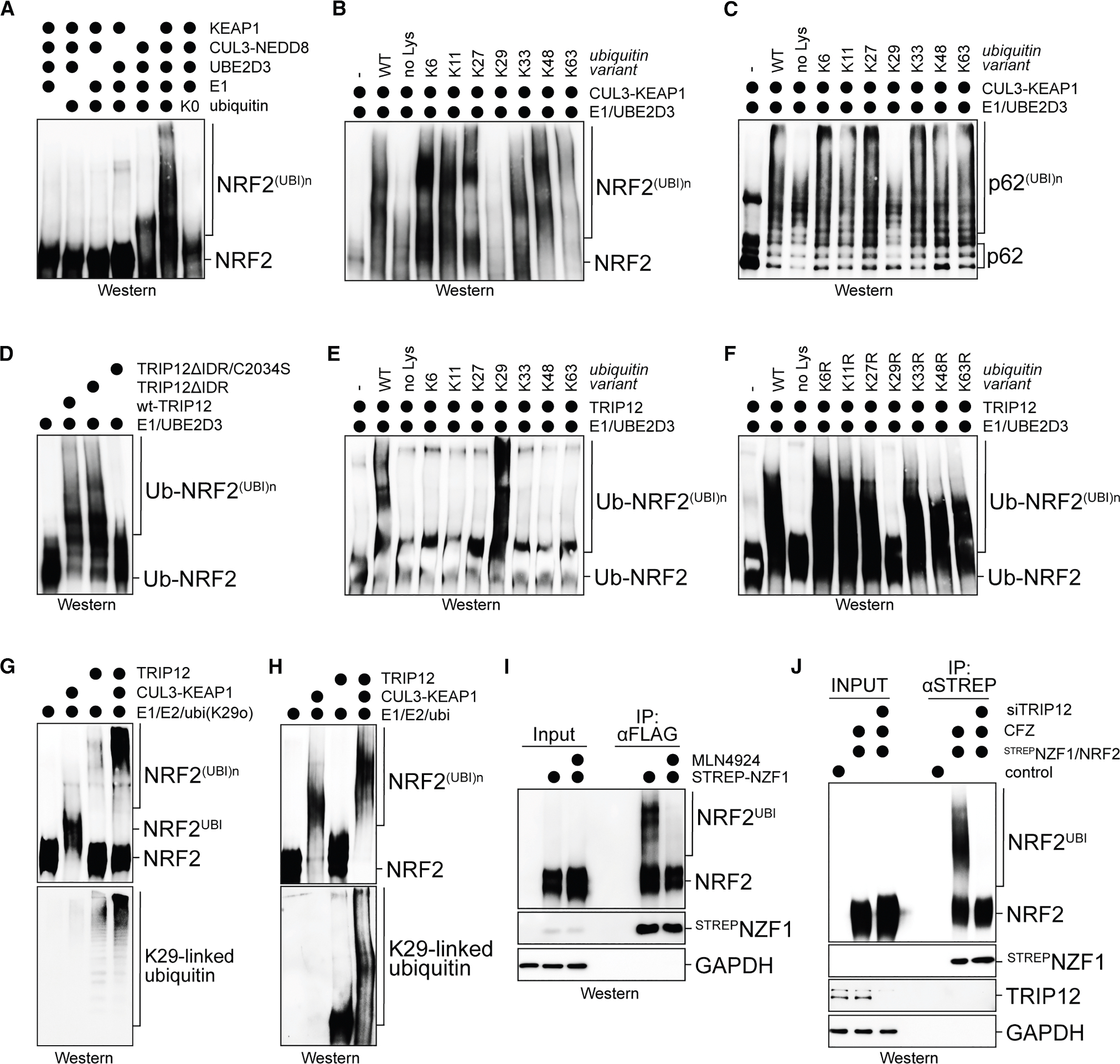
TRIP12 is a ubiquitin chain elongation factor for NRF2 (A) Ubiquitylation of NRF2 by NEDD8-modified CUL3^KEAP1^ in the presence of wild-type (WT) ubiquitin or a ubiquitin variant containing no Lys residues (ubi-K0). Reaction products were analyzed by western blotting. (B) Ubiquitylation of NRF2 by CUL3^KEAP1^ and UBE2D3 in the presence of ubiquitin mutants that contained only a single Lys residue (K6: all Lys residues, except K6, mutated to Arg). Reaction products were analyzed by western blotting. (C) Ubiquitylation of p62 by CUL3^KEAP1^ and UBE2D3 in the presence of ubiquitin mutants that contained only a single Lys residue. Reaction products were analyzed by western blotting. (D) Ubiquitylation of Ub~NRF2 by TRIP12, a TRIP12 variant lacking its N-terminal intrinsically disordered region (TRIP12ΔIDR), or a TRIP12ΔIDR variant that also had its catalytic Cys residue mutated. Reaction products were analyzed by western blotting. (E) Ubiquitylation of Ub~NRF2 by TRIP12 in the presence of ubiquitin mutants containing a single Lys residue. Reaction products were analyzed by western blotting. (F) Ubiquitylation of Ub~NRF2 by TRIP12 in the presence of ubiquitin mutants that lacked a single Lys residue (K6R: only Lys6 of ubiquitin mutated to Arg). Reaction products were analyzed by western blotting. (G) Ubiquitylation of NRF2 by CUL3^KEAP1^, TRIP12, or both, using a ubiquitin variant containing K29 as its only Lys residue. Reaction products were analyzed by western blotting using antibodies against NRF2 and K29-linked ubiquitin chains. (H) Ubiquitylation of ^HIS^NRF2 by CUL3^KEAP1^, TRIP12, or both in the presence of WT ubiquitin. ^HIS^NRF2 was purified from reaction mixtures and analyzed for ubiquitylation by western blotting using antibodies against NRF2 or K29-linked ubiquitin chains. (I) C2C12 myoblasts expressed NRF2 as well as a Streptavidin (Strep)-tagged NZF1 domain of the K29/K33-specific deubiquitylase TRABID. ^STREP^NZF1 was affinity purified from lysates under stringent conditions, and co-precipitating NRF2 was detected using αNRF2 antibodies. Where indicated, MLN4924 was added to prevent the chain initiation event required for TRIP12-dependent chain elongation. (J) C2C12 myoblasts expressed NRF2 as well as a Strep-tagged NZF1 domain of the K29/K33-specific DUB TRABID. As indicated, TRIP12 was depleted by siRNAs. ^STREP^NZF1 was affinity purified from lysates under stringent conditions, and co-precipitating NRF2 was detected using αNRF2 antibodies. See also Figure S3.

Reducing TRIP12 levels alleviated the effects of partial NRF2 depletion on myogenesis (Figure S3A), which is expected if TRIP12 were to modulate the stability of any NRF2 that is remaining in siRNA-treated cells. To test whether TRIP12 targets NRF2, we reconstituted the ubiquitylation of NRF2 using purified components. We incubated NRF2 with CUL3^KEAP1^ and the E2 UBE2D3, which is often used in studies with CUL3 E3 ligases,^31,32^ and found that this led to decoration of NRF2 with ubiquitin polymers (Figure 4A). When we performed this reaction with ubiquitin variants containing a single lysine, we noted that CUL3^KEAP1^ and UBE2D3 possessed little linkage specificity, except that they failed to produce K29-linked conjugates (Figure 4B). Also, with p62 as substrate, CUL3^KEAP1^ and UBE2D3 did not build polymers if K29 was the only available lysine (Figure 4C), and a linkage-specific ubiquitin antibody showed that CUL3^KEAP1^ and UBE2D3 did not modify NRF2 with K29-linkages using wild-type ubiquitin (Figure S3B). Conversely, CUL3^KEAP1^ and UBE2D3 were more efficient in polyubiquitylating NRF2 if K29 had been mutated (Figure S3C). When collaborating with a related E2, UBE2D2, CUL3^KEAP1^ multimonoubiquitylated NRF2 rather than building polymers (Figure S3D), while other E2 enzymes were inactive or even impaired the ability of CUL3^KEAP1^ to use UBE2D3 (Figures S3E–S3G). As a consequence of this linkage specificity, every ubiquitin attached to NRF2 by CUL3^KEAP1^ and UBE2D3 or UBE2D2 has K29 available for further modification.

TRIP12 by itself did not modify NRF2 with ubiquitin chains of high molecular weight (MW) (Figure S3H). As TRIP12 had been linked to the ubiquitin-fusion pathway,^74,75^ we asked whether it extends ubiquitin conjugates that had already been attached to NRF2 by distinct enzymes, such as CUL3^KEAP1^. We therefore incubated TRIP12 with NRF2 that was fused to ubiquitin to bypass the initiation step (Ub~NRF2) and found that TRIP12 efficiently modified this protein (Figures 4D and S3H). The ability of TRIP12 to target Ub~NRF2 required the catalytic Cys of the HECT domain, but not an intrinsically disordered region at its N terminus (Figure 4D). Performing this experiment with ubiquitin variants revealed that K29 of ubiquitin was required and sufficient for modification of Ub~NRF2 by TRIP12 (Figures 4E and 4F), and mutating K29 in the fused ubiquitin abolished TRIP12 activity toward Ub~NRF2 (Figure S3H). As seen for other substrates,^71^ TRIP12 therefore extends K29-linked conjugates on Ub~NRF2, a specificity that is complementary to the linkage exclusivity of CUL3^KEAP1^. Given that TRIP12 usually engages NRF2 through CUL3, its ability to decorate Ub~NRF2 with ubiquitin polymers is likely due to chain elongation factors binding ubiquitin,^76^ an ability also detected for TRIP12 (Figure S3I).

Based on these findings, we asked if CUL3 and TRIP12 together ubiquitylate NRF2 more efficiently than either enzyme alone. We incubated NRF2 with ubi-K29 and CUL3^KEAP1^ to allow for chain initiation, but not elongation, and then added TRIP12. Reflecting their complementary linkage specificities, only the combination of CUL3^KEAP1^ and TRIP12 stimulated formation of high-MW conjugates, and K29 linkages were detected only when TRIP12 had been included in the reaction (Figure 4G). We next performed this experiment using wild-type ubiquitin and repurified NRF2 for analysis with linkage-specific antibodies.^77^ This strategy confirmed that CUL3^KEAP1^ did not produce detectable K29-linked conjugates on NRF2, underscoring its intriguing linkage exclusivity (Figure 4H). The combination of CUL3^KEAP1^ and TRIP12 resulted in polymers of higher MW, and K29 linkages were detected only if TRIP12 was present. In line with these results, we found that NRF2 was modified in cells with conjugates that were enriched by a K29/K33-linkage-specific ubiquitin-binding domain (Figure 4I). The K29-specific ubiquitylation of NRF2 was prevented by MLN4924, as expected due to MLN4924 inhibiting the first step of CUL3^KEAP1^/TRIP12-dependent modification. In addition, K29-linkage-specific modification of NRF2 was obliterated upon depletion of TRIP12 (Figure 4J). We conclude that TRIP12 is a chain elongation factor that cooperates with CUL3^KEAP1^ to decorate NRF2 with K29-linked ubiquitin chains that drive efficient proteasomal degradation during stress.^71,78^

### TRIP12 restrains NRF2 accumulation

**Figure 5.**
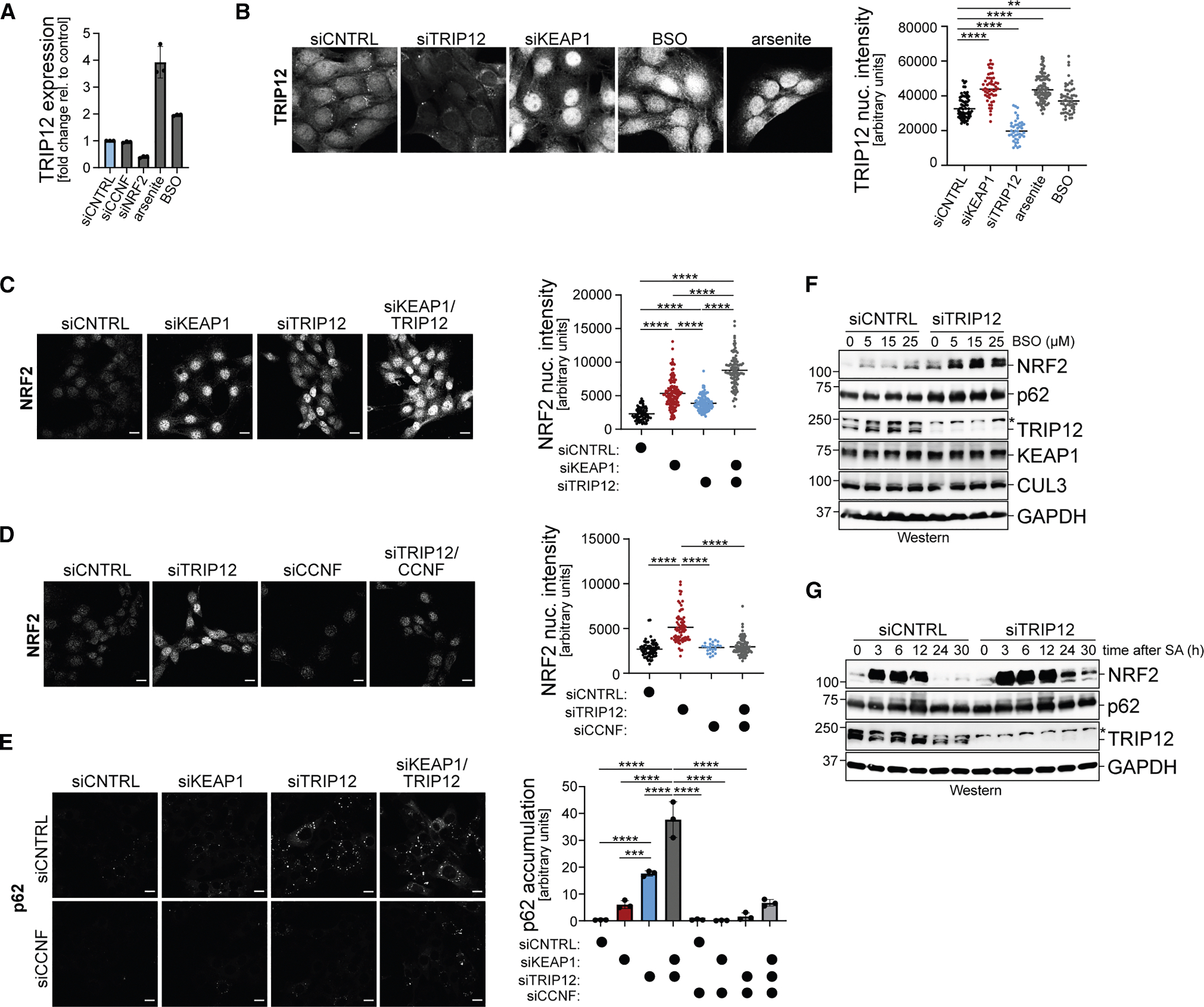
TRIP12 restricts accumulation of CUL3^KEAP1^ substrates (A) RT-qPCR analyses of TRIP12 mRNA levels upon oxidative stress imposed by sodium arsenite or BSO or upon NRF2 depletion. (B) Nuclear localization of endogenous TRIP12 increases upon oxidative stress. TRIP12-depleted cells served as negative control. Quantification is shown on the right. Data are represented as the mean ± standard deviation. **p < 0.01 and ****p < 0.0001. (C) Depletion of KEAP1, TRIP12, or both in C2C12 myoblasts results in accumulation of nuclear NRF2, as determined by immunofluorescence against endogenous NRF2. Quantification is shown on the right. Data are represented as the mean ± standard deviation. ****p < 0.0001. Scale bars, 10 μm. (D) Accumulation of nuclear NRF2 in C2C12 myoblasts upon TRIP12 depletion is lost by simultaneous inactivation of CCNF, as determined by microscopy against endogenous NRF2. Quantification is shown on the right. Data are represented as the mean ± standard deviation. ****p < 0.0001. Scale bars, 10 μm. (E) Depletion of KEAP1 or TRIP12 in C2C12 myoblasts results in accumulation of p62 puncta, as shown by microscopy against endogenous p62. Co-depletion of CCNF reverts effects of KEAP1 or TRIP12 loss. Quantification is shown on the right. Data are represented as the mean ± standard deviation. ***p < 0.001 and ****p < 0.0001. Scale bars, 10 μm. (F) Depletion of TRIP12 in C2C12 myoblasts stabilizes NRF2 in cells exposed to increasing concentrations of the oxidative stressor BSO, as shown by western blotting. (G) Depletion of TRIP12 in C2C12 myoblasts delays degradation of NRF2 after recovery from sodium arsenite-induced oxidative stress (25 μM for indicated times), as shown by western blotting. See also Figure S4.

Gene expression analyses revealed that TRIP12 mRNA levels increased if cells were exposed to oxidative stressors, while they were lowered by depletion of NRF2 (Figure 5A). The increase in *TRIP12* expression upon oxidative stress occurred at times when NRF2 levels peaked (Figure S4A). While less dramatic, TRIP12 protein levels also increased concomitant with NRF2 stabilization during stress (Figure S4B), and TRIP12 accordingly accumulated in the nucleus under such conditions (Figure 5B). These observations implied that TRIP12 may function late during oxidative stress, when ROS are being cleared and cells need to restore NRF2 turnover.

We therefore asked whether TRIP12 limits the accumulation of NRF2 in the nucleus, where the transcription factor accumulates upon stress. Indeed, despite weaker effects than seen with lowering KEAP1, depletion of TRIP12 increased the abundance of nuclear NRF2 (Figure 5C). Targeting KEAP1 and TRIP12 at the same time led to a further accumulation of nuclear NRF2 (Figure 5C). While this effect is likely due to incomplete depletion by siRNAs, it highlights that TRIP12 supports the degradation of NRF2 likely in conjunction with CUL3^KEAP1^.

The consequences of TRIP12 depletion onto NRF2 accumulation were specific: neither loss of UBR5, which is correlated with TRIP12 on DepMap and known to target transcription factors,^66,79^ nor depletion of E3 ligases detected in some NRF2 immunoprecipitations affected NRF2 levels in the nucleus (Figures S4C and S4D). By contrast, lowering CCNF blunted the increase in nuclear NRF2 in either TRIP12- or KEAP1-depleted cells (Figures 5D and S4E), which underscores that TRIP12 and KEAP1 act in the same pathway to regulate NRF2. In line with these findings, depletion of TRIP12 reduced ROS levels in myoblasts, which was counteracted by concomitant loss of NRF2 or CCNF (Figures S4F and S4G).

In addition to stabilizing NRF2, CUL3^KEAP1^ inhibition caused formation of cytoplasmic p62 foci (Figure 5E). Lowering TRIP12 also increased the abundance of p62 foci and, in fact, had a more pronounced effect than loss of KEAP1 (Figure 5E). As seen for NRF2, co-depleting KEAP1 and TRIP12 led to greater accumulation of p62 foci than either siRNA alone. Thus, TRIP12 also sustains regulation of p62, a protein that can shuttle between cytoplasm and nucleus.^80^ Loss of CCNF counteracted the effects of KEAP1 or TRIP12 depletion on the formation of p62 foci, which is in line with our observation that TRIP12 binds CUL3 and thus can encounter multiple substrates presented through KEAP1.

Its induction during oxidative stress implied that TRIP12 might be important at times when ROS are being cleared. To address this question, we had to detect effects of TRIP12 depletion on NRF2 by western blotting, which is less sensitive than microscopy but can easily be used to compare multiple conditions. We first depleted myoblasts of TRIP12 before treating cells with increasing concentrations of BSO, hydrogen peroxide, or sodium arsenite. While these oxidative stressors caused the expected increase in NRF2 levels, this was more pronounced when TRIP12 had been depleted (Figures 5F, S4H, and S4I). As arsenite elicits transient stress,^55^ we used this compound to test if TRIP12 helps cells to effectively downregulate NRF2 again, as stress is being resolved. In control cells, NRF2 accumulated upon exposure to arsenite and was degraded again as cells were recovering (Figure 5G). By contrast, NRF2 persisted much longer in TRIP12-depleted cells (Figure 5G), showing that TRIP12 supports NRF2 degradation as cells recover from oxidative stress and NRF2 needs to be turned over to prevent reductive stress.

### TRIP12 restricts oxidative stress signaling

**Figure 6.**
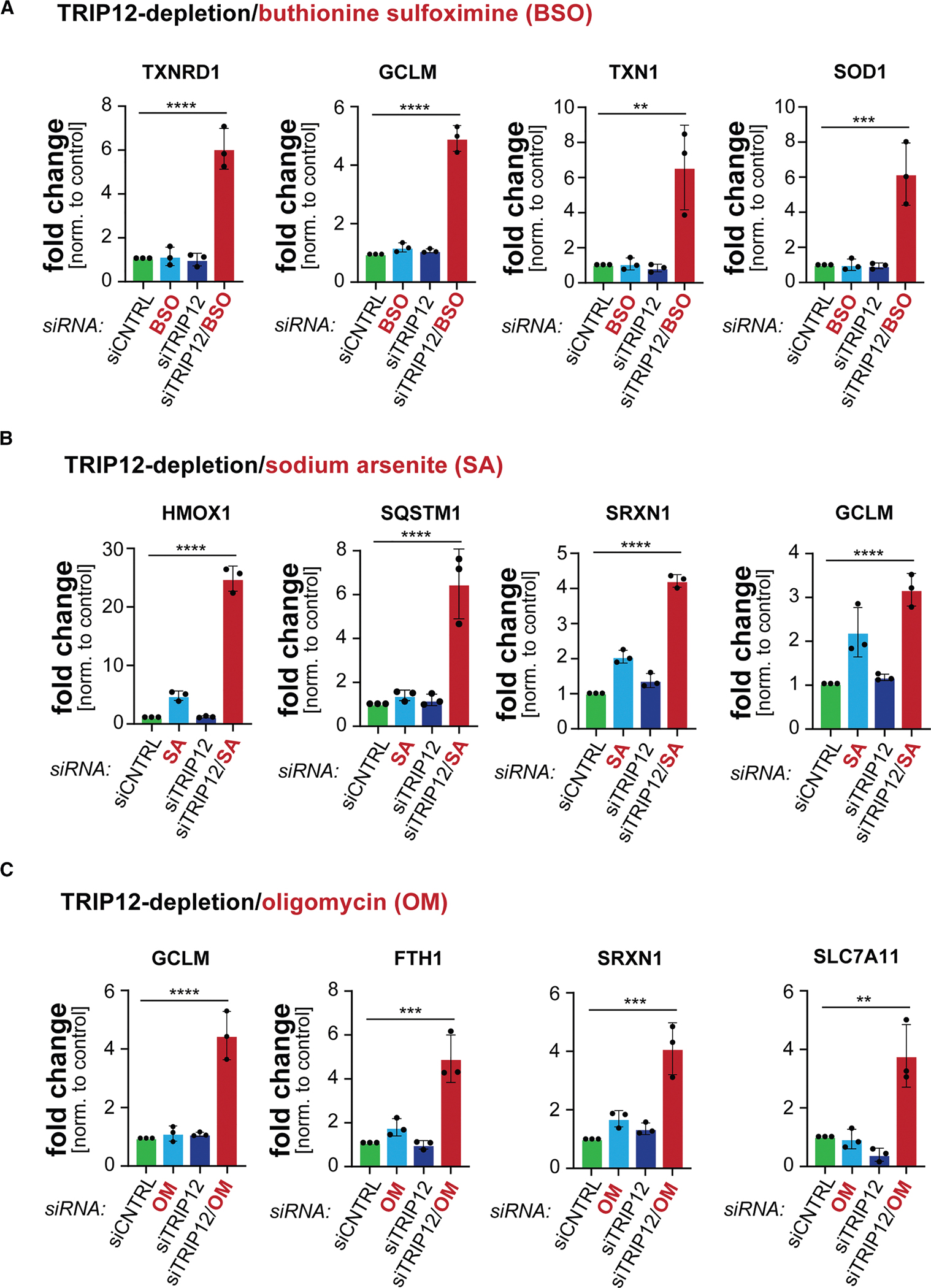
TRIP12 restricts antioxidant gene expression (A) C2C12 myoblasts were treated with the glutathione synthesis inhibitor BSO (16 h; 5 μM), depleted of TRIP12, or both. Expression of NRF2 target genes was determined by qPCR. *n* = 3 replicates. Data are represented as the mean ± standard deviation. **p < 0.01, ***p < 0.001, and ****p < 0.0001. (B) C2C12 myoblasts were treated with sodium arsenite (16 h; 5 μM), depleted of TRIP12, or both. Expression of NRF2 target genes was determined by qPCR. *n* = 3 replicates. Data are represented as the mean ± standard deviation. ****p < 0.0001. (C) C2C12 myoblasts were treated with the mitochondrial ATP synthase inhibitor oligomycin (16 h; 0.9 μM), depleted of TRIP12, or both. Expression of NRF2 target genes was determined by qPCR. *n* = 3 replicates. Data are represented as the mean ± standard deviation. **p < 0.01, ***p < 0.001, and ****p < 0.0001.

Having seen that TRIP12 ensures proper NRF2 degradation, we wished to determine if it impacts antioxidant gene expression. Partial inactivation of TRIP12, as accomplished by siRNAs, was not sufficient to elicit a pronounced spike in the expression of NRF2 targets (Figures 6A–6C). In a similar manner, moderate concentrations of BSO, sodium arsenite, or oligomycin did not trigger a persistent oxidative stress response. However, if TRIP12-depleted cells were exposed to the same oxidative insults, many NRF2 targets were strongly induced (Figures 6A–6C). Depletion of TRIP12 increased the expression of NRF2 targets relevant to the underlying biology: for example, BSO-dependent inhibition of glutathione synthesis in TRIP12-depleted cells resulted in strong upregulation of the GCLM subunit of glutamate-cysteine ligase, while arsenite-dependent induction of oxidative and proteotoxic stress led to pronounced expression of p62 as a regulator of autophagy (Figures 6A–6C). These experiments showed that TRIP12 restricts NRF2 activation, which was particularly noticeable if cells had experienced oxidative stress.

**Figure 7.**
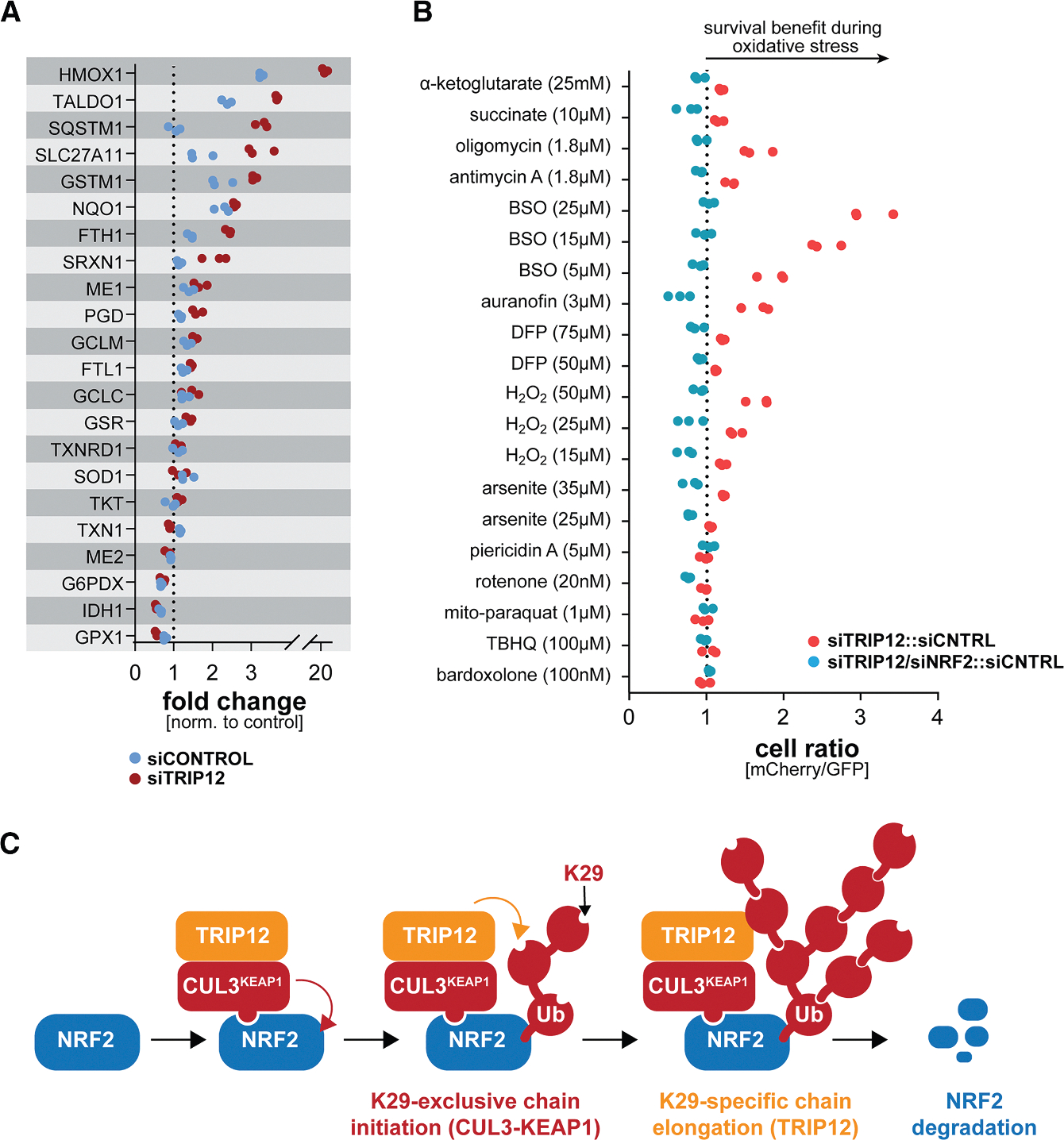
TRIP12 restricts oxidative stress signaling and cell survival during stress (A) C2C12 myoblasts were transfected with control siRNAs or siRNAs targeting TRIP12. Oxidative stress was induced by exposing cells to sodium arsenite (24 h; 25 μM). After 24 h, NRF2 target gene expression was determined by qPCR. (B) GFP-labeled control cells were mixed at a 1:1 ratio with mCherry-labeled cells depleted of TRIP12 (red dots). As indicated, NRF2 was depleted (blue dots). Cells were exposed to increasing concentrations of oxidative stressors. After 2 days, the ratio of GFP- to mCherry-labeled cells was determined by flow cytometry. *n* = 3 independent experiments. (C) Model of NRF2 ubiquitylation carried out by CUL3^KEAP1^ and TRIP12. See also Figure S5.

To investigate if TRIP12 helps to shut down antioxidant gene expression during recovery from stress, we treated cells with a pulse of sodium arsenite and monitored mRNA levels at times when NRF2 has usually been degraded (see Figure 5E). While control cells shut down NRF2 targets at this time, cells depleted of TRIP12 continued to express antioxidant genes and hence showed a delay in silencing the oxidative stress response (Figure 7A). Thus, TRIP12 accelerates the return of cells to homeostatic conditions by shutting off NRF2-dependent gene expression.

As TRIP12 helps cells to rein in NRF2, we finally asked if TRIP12 inhibition would boost cell survival during oxidative stress, when NRF2 accumulation is temporarily beneficial. Indeed, depletion of TRIP12 had the same benefits as reducing KEAP1 and improved myoblast survival upon glutathione synthesis inhibition by BSO, TXNRD1 inhibition by auranofin, ATPase inhibition by oligomycin, or exposure to H_2_O_2_ (Figure 7B). As seen for KEAP1, the protective effect of lowering TRIP12 was dependent on NRF2 (Figure 7B), and it required both CCNF and p62 (Figures S5A and S5B). We conclude that TRIP12 regulates the oxidative stress response by restricting duration and extent of NRF2 activation. Our work also revealed that the need for dynamic control of the oxidative stress response comes at a cost: to ensure that ROS clearance is quickly followed by a return to homeostatic conditions, cells degrade some NRF2 even during stress despite this limiting their ability to survive challenging conditions.

## DISCUSSION

Dynamic control of oxidative stress signaling is critical for cell and tissue homeostasis. When cells detect ROS, they stabilize NRF2 to induce antioxidant gene expression. However, as cells overcome oxidative insults, they must eliminate NRF2 to prevent reductive stress and its deleterious consequences on cellular metabolism and function. Here, we show that the dynamic regulation of NRF2 stability and antioxidant signaling requires TRIP12, an essential E3 ligase that is dysregulated in Parkinson’s disease, a pathology that is characterized by aberrant ROS management. TRIP12 elongates CUL3^KEAP1^-dependent ubiquitin conjugates to ensure robust NRF2 turnover (Figure 7C). In this manner, TRIP12 restricts NRF2 activation during stress, but facilitates NRF2 elimination during recovery. The ability of cells to ensure transient activation of the oxidative stress response is therefore so important that it comes at the cost of limited protective signaling in the face of ROS.

### Mechanism of NRF2 ubiquitylation

Even though CUL3^KEAP1^ has long been known to be an E3 ligase for NRF2, several observations indicated that additional factors might be required for robust NRF2 turnover. As one such protein, we identify TRIP12 as a chain elongation factor, also referred to as E4,^81^ that amplifies ubiquitin conjugates attached to NRF2 by CUL3^KEAP1^. While we discovered this role of TRIP12 in myoblasts, DepMap analyses indicate that TRIP12 and CUL3^KEAP1^ similarly work together in other cell types. TRIP12 also functions in the ubiquitin-fusion pathway,^74,75^ and it collaborates with CUL2 and CUL4 E3 ligases to jump-start targeted protein degradation.^71^ It therefore appears that TRIP12 amplifies the ubiquitin signals of multiple Cullin-based E3 ligases. In line with such a central function, *TRIP12* is essential for mouse development,^49^ and its products, K29-linked ubiquitin conjugates, are abundant in human cells.^77^

Our proteomic analyses showed that TRIP12 interacted less efficiently with an NRF2 variant lacking its KEAP1 degron, which implied that TRIP12 binds NRF2 via CUL3^KEAP1^. Indeed, TRIP12 bound CUL3 strongly and directly. Formation of stable complexes between TRIP12 and CUL3^KEAP1^ likely facilitates efficient ubiquitin handover from the initiation site in CUL3^KEAP1^ to the elongation module provided by TRIP12, thereby promoting robust NRF2 degradation (Figure 7C). Similar ubiquitin handover has recently been observed for SIFI, a large E3 ligase that mediates silencing of a mitochondrial stress response.^82^

Biochemical experiments revealed that TRIP12 and CUL3^KEAP1^ possess linkage complementarity. CUL3^KEAP1^ and its preferred E2 UBE2D3 did not modify K29 of ubiquitin, a catalytic preference that we refer to as linkage exclusivity. Why CUL3^KEAP1^ inefficiently targets K29 of ubiquitin is unclear, but it might bind ubiquitin in a manner that orients this lysine away from the E2 enzyme. When acting with UBE2D2, CUL3^KEAP1^ catalyzed multimonoubiquitylation. With both E2s, CUL3^KEAP1^ therefore assembles conjugates in which many building blocks have K29 of ubiquitin available for chain elongation, setting up efficient chain elongation by the K29-specific TRIP12. We anticipate that TRIP12 acts on multiple ubiquitin subunits on each NRF2, thereby producing polymers that resemble branched chains for efficient proteasomal degradation.^83^ Branched chains recruit the segregase p97/VCP,^83–85^ which is required for NRF2 degradation.^86^ In DepMap, TRIP12 is correlated with the p97 interactors PLAA and VCPIP,^66^ and its products, K29-linked conjugates, co-localize with p97 in stressed cells.^77^ Together, these findings suggest that CUL3^KEAP1^ and TRIP12 together produce a ubiquitin signal that enables NRF2 turnover if cells need to return to homeostatic conditions during recovery.

### Role of TRIP12-dependent ubiquitin chain extension

Underscoring the relevance of TRIP12 for NRF2 regulation, its depletion unleashed antioxidant gene expression if cells experienced moderate oxidative stress and CUL3^KEAP1^ had been compromised. Moreover, inactivation of TRIP12 delayed silencing of NRF2-dependent transcription once ROS had been cleared. TRIP12-dependent amplification of ubiquitin signals is therefore particularly important when CUL3^KEAP1^ is less active and fewer chain initiation events are expected to occur. How could adding more ubiquitin molecules to NRF2 improve proteasomal degradation? We propose that, without much linkage specificity, the ubiquitin polymers produced by CUL3^KEAP1^ are poor proteasomal targeting signals. Attachment of K29-linked conjugates by TRIP12 adds ubiquitin chains that improve recognition of NRF2 by p97 and the proteasome to increase the efficiency of degradation. As the flip side of this regulation, if chain initiation and elongation are poorly coordinated or TRIP12 is not expressed, proteins bound to CUL3^KEAP1^ might escape proteasomal degradation.

Depletion of TRIP12 by siRNAs had weaker effects on NRF2 accumulation and function than lowering KEAP1. Thus, CUL3^KEAP1^ likely controls the rate-limiting step in NRF2 ubiquitylation, and TRIP12 predominantly becomes critical when cells need to return to homeostatic conditions. Alternatively, other chain elongation factors might support ubiquitin chain formation when TRIP12 has been depleted. It is important to note that we could not generate *ΔTRIP12* myoblasts and hence had to rely on siRNAs that partially deplete their target. While our failure to obtain *ΔTRIP12* myoblasts is consistent with TRIP12’s essential role in embryogenesis, it raises the possibility that the remaining TRIP12 in siRNA-treated cells is sufficient to accomplish sufficient NRF2 ubiquitylation.

By depleting KEAP1 or TRIP12, we noticed that cells continue to degrade some NRF2 even if they experience strong oxidative stress. While the residual NRF2 turnover restricts protective signaling and limits cell survival, it eases the return of cells to homeostatic conditions as ROS are being cleared. A failure to shut off NRF2 depletes ROS that are required for cell differentiation and tissue homeostasis.^14,15,17,87–90^ Recent work showed that such ROS are sentinel molecules that allow cells to fine-tune the activity of their electron transport chain that is required for metabolic function and cell differentiation.^91^ Moreover, prolonged KEAP1 inhibition results in a form of cell death referred to as oxeiptosis,^20^ and inactivation of CUL3^KEAP1^ by mutations in *KEAP1* or *CUL3* drives multiple cancers.^7,22,92^ The importance of establishing transient stress response activation has been documented beyond NRF2, such as for mitochondrial and proteotoxic stress responses.^55,93,94^ Together with these findings, our discovery of TRIP12 as a negative regulator of the oxidative stress response underscores that dynamic regulation of stress signaling is critical for preserving organismal health.

We were prompted to search for additional NRF2-E3 ligases after having observed the effects of CCNF depletion on myoblast differentiation. Lowering CCNF stimulated proteasomal turnover of NRF2 even if KEAP1 had been depleted, which prevented antioxidant gene expression and restored the ability of myoblasts to differentiate.^15^ How CCNF impacts NRF2 stability is unknown, but RNA sequencing showed that CCNF depletion rewires gene expression, consistent with previously reported effects on cell-cycle progression.^51,53,54,56^ Cells lacking CCNF accumulate in S and G2, the same cell-cycle stages when TRIP12 levels increase.^50^ We therefore propose that CCNF depletion stimulates NRF2 turnover in part by increasing TRIP12 production, but more work is needed to test this hypothesis.

### Therapeutic potential of TRIP12

As ROS accumulation is a hallmark of neurodegenerative diseases,^1,95^ transient NRF2 stabilization by small molecules might be of therapeutic benefit. While KEAP1 inhibition would be one approach to induce NRF2, it is dangerous: mutations in *KEAP1* or in the KEAP1-binding motif of NRF2 cause cancer.^92^ *TRIP12* variants have been observed in cancer but are unlikely to be driver mutations. We suggest that TRIP12 inhibition provides a safer route to activating NRF2 without promoting tumorigenesis. At least in myoblasts, TRIP12 inhibition offers the same survival benefit during stress as loss of KEAP1 but preferentially increases NRF2 levels if cells experience some ROS, as expected for neurons at the brink of degeneration. Our work therefore reveals a component of the oxidative stress response, TRIP12, that could be targeted to rewire this protective signaling system for therapeutic benefit.

### Limitations of the study

While this study deepens our understanding of the oxidative stress response, a pathway linked to cancer and neurodegeneration, it raises several questions. How CCNF modulates NRF2 stability is unknown: does CCNF regulate the CUL3^KEAP1^-TRIP12 machinery, potentially through its role in cell-cycle control,^51^ or does it act through an additional E3 ligase? A technical limitation of our work is that *TRIP12* is essential for embryogenesis, and we failed to delete it from myoblasts. Given that we had to rely on depletion of TRIP12 by siRNAs, it is unclear if TRIP12 is the only chain elongating factor for NRF2 or whether additional enzymes help establish silencing of the oxidative stress response. Finally, given the strong interaction between TRIP12 and CUL3, it will be important to determine whether other CUL3 E3 ligases, such as CUL3^SPOP^, rely on chain elongation by TRIP12. Given the roles of CUL3^KEAP1^ and CUL3^SPOP^ in tumorigenesis, dissecting the mechanisms of ubiquitin chain formation by these enzymes is an important area of study that holds promise for developing novel therapeutic approaches.

## RESOURCE AVAILABILITY

### Lead contact

Requests for further information, reagents, and resources should be directed to Michael Rapé (mrape@berkeley.edu).

### Materials availability

Plasmids generated in this study will be deposited on Addgene and are also available from the lead contact upon request. No gene-edited cell lines or mouse lines were generated in this study.

### Data and code availability

- Original RNA sequencing data have been deposited into the NCBI Gene Expression Omnibus (GEO: GSE281184). Original mass spectrometry data have been deposited into the MassIVE database (MSV: 000098529). It is accessible using this link: https://massive.ucsd.edu/ProteoSAFe/dataset.jsp?accession=MSV000098529. Please see the key resources table. Additional information to reanalyze data reported in this work paper is available from the lead contact upon request.
- Code availability: no code has been developed in this work.
- Other items availability: all plasmids generated in this work can be requested from the lead contact’s lab and will be freely shared. All antibodies, chemicals, and cell lines used in this study are commercially available.

## STAR★METHODS

### EXPERIMENTAL MODEL AND STUDY PARTICIPANT DETAILS

#### Cell lines

All cell lines were purchased from ATCC. C2C12 myoblasts and HEK293T cells were grown in DMEM + glutamax (GIBCO, 51985091) with 10% fetal bovine serum (VWR, 97068–107). For C2C12 differentiation experiments, cells were grown to 70–90% confluence and media was changed into DMEM + glutamax (GIBCO, 51985091) with 2% fetal bovine serum (VWR, 97068–107). Media was changed daily for 3–4 days until cells differentiated. All cell lines regularly tested negative for mycoplasma.

### METHOD DETAILS

#### Cell culture

C2C12 myoblasts and HEK293T cells were grown in DMEM + glutamax (GIBCO, 51985091) with 10% fetal bovine serum (VWR, 97068–107). C2C12 myoblasts were differentiated by changing media to DMEM + glutamax (GIBCO, 51985091) with 2% fetal bovine serum (VWR, 97068–107) daily for 3–4 days. siRNA transfections were performed with Lipofectamine RNAiMAX (Thermo Fisher Scientific, 13778030) according to manufacturer’s instructions. In C2C12 myoblasts, siRNAs were used at a final concentration of 30 nM. For experiments using western blotting or qPCR, cells were harvested 48h after siRNA transfection. All other transfections were done using Lipofectamine 3000 (Thermo Fisher Scientific, L3000008) according to manufacturer’s instructions.

#### Virus production and infection

Lentivirus for pLVX-2xStrep-CUL3-IRES-Puro, pLVX-GFP-P2A-Blast, pLVX-mCherry-P2A-blast, pLVX-3xFLAG-NRF2-IRES-Puro, and pLVX-3xFLAG-NRF2ΔETGE-IRES-Puro were made by co-transfection in HEK293T of viral plasmids with packaging plasmids using lipofectamine 3000 (Thermo Fisher Scientific, L3000008). Virus containing supernatants were collected 72h after transfection, spun down to remove any remaining cells, and concentrated in LentiX Concentrator (Takara, 631262) according to manufacturer’s instructions. After concentrating, virus was aliquoted and stored at −80°C for further use. To infect C2C12 myoblasts with virus, 50000 cells were seeded in a 12-well plate, mixed with virus and 6μg/ml polybrene (Sigma-Aldrich, TR-1003), and centrifuged for 45min at 1000g. Selection was started 24h later with either puromycin (1.5μg/ml, Sigma-Aldrich, P8833) or blasticidin (10μg/ml, Thermo Fisher Scientific, A1113903).

#### Immunoprecipitation and mass spectrometry

HEK293T cells were infected with pLVX-3xFLAG-NRF2-IRES-Puro or pLVX-3xFLAG-NRF2ΔETGE-IRES-Puro virus as described above. Cells were seeded in ten 15cm plates per condition and grown to 80–90% confluence. MLN4924 (500 nM Selleckchem, S7109) was added 16h before harvesting. Cells were lysed in 20 mM HEPES pH 7.5, 150 mM NaCl, 0.2% Nonidet P-40 (Sigma-Aldrich, E1014, 1x complete protease inhibitor cocktail (Roche, 11836170001), for 30 minutes at 4°C, clarified by centrifuging for 15min at 18000g at 4°C, and incubated with αFLAG M2 resin (Sigma-Aldrich, A2220) for 2h at 4°C. After incubation, resin was washed 3 times in lysis buffer and proteins were eluted with 0.5mg/ml 3xFLAG peptide (Sigma-Aldrich, F4799) buffered in 1x PBS, 0.1% triton X-100. Eluted proteins were precipitated overnight at 4°C by adding trichloroacetic acid to a concentration of 20%. Protein pellets were centrifuged and washed three times in acetone with 0.1M HCl, then dried at room temperature, and resuspended in 8M urea with 100mM Tris, pH8.5. Samples were reduced by adding 5mM TCEP (Sigma-Aldrich, C4706) for 20min, alkylated with 10mM iodoacetamide (Thermo Fisher Scientific, A39271) for 15min, diluted four-fold in 100mM Tris, pH8.5, and digested with 0.5mg/ml trypsin (Promega, v5111) with 1 mM CaCl_2_ overnight at 37°C while shaking. Samples were analyzed at the UC Berkeley Vincent J. Coates Proteomics and Mass Spectrometry Laboratory using multidimensional protein identification technology (MudPIT) and run on a LTQ XL linear ion trap mass spectrometer. High confidence interactors were identified using ComPASS to compare the identified peptides with numerous unrelated mass spectrometry samples from our laboratory ^99^. These high confidence interactors are presented in Figure 3D. Samples were normalized to the total spectral counts (TSC) of the NRF2 bait peptides. Complete proteomics data of all identified peptides is provided in Table S2.

#### Small-scale immunoprecipitation

Cells were grown in 1–3 15cm plates per condition and lysed as described. After clearing lysate by centrifugation, samples were normalized using Pierce 660 nm Protein Assay Reagent (Thermo Fisher Scientific, 22660). After normalization, 2.5% of the sample was saved as input and samples were incubated with either αFLAG M2 resin (Sigma-Aldrich, A2220) for KEAP1^FLAG^ pull-down or Strep-Tactin XT 4Flow resin (IBA, 2–5010-025) for ^Strep^CUL3 or Strep-NZF1 pull-downs at 4°C for 1h. Samples were washed three times in lysis buffer and eluted in twice in 120mM Tris, pH6.8, 4%SDS, M urea, 20% glycerol, 5% β-mercaptoethanol, bromophenol blue. SDS-PAGE and immunoblotting were performed using the indicated antibodies and images were captured using a ProteinSimple FluorChem M device.

#### Western blotting from whole cell lysates

Whole cell lysates were prepared by harvesting cells in lysis buffer as described above, but with the addition of 1μl Benzonase (EMD Millipore, 70746–4) per ml lysis buffer. Samples were incubated on ice for 15min and normalized as using Pierce 660 nm Protein Assay Reagent (Thermo Fisher Scientific, 22660). Samples were combined with 2x urea sample buffer and analyzed using SDS-PAGE and Western blotting using primary antibodies. Secondary antibodies conjugated to HRP were added after washing in 1x PBS with 0.1% Tween. Membranes were incubated for 2h, washed, and imaged by incubating with Immobilon Western Chemiluminescent Substrate (Millipore, WBKLS05000) according to manufacturer’s instructions and capturing images with a ProteinSimple FluorChem M device.

#### Recombinant DNA

Constructs were cloned into pCS2+, pLVX, pET28a, or pFastBac plasmid backbones using Gibson assembly using HIFI DNA Assembly master mix (NEB, E2621L) after amplification by PCR using PrimeSTAR GXL DNA Polymerase (Takara, R050A) of the gene of interest out of cDNA prepared from HEK293T cells using the Protoscript II First Strand cDNA Synthesis Kit (NEB, E6560S).

#### Antibodies

The following antibodies were used in this study: αNRF2 (Cell Signaling, 12721S), α KEAP1 (Cell Signaling, 7705S), αTRIP12 (Proteintech, 25303–1-AP), αSQSTM1 (Abcam, ab56416), αGAPDH (Cell Signaling, 14C10), α-β-actin (MP Biomedicals, 08691001, clone C4), αmyosin heavy chain, sarcomere (MHC) (mouse monoclonal; Developmental Studies Hybridoma Bank, clone MF20), αCUL3 (mouse monoclonal; generated with Covance ^100^), StrepMAB-Classic αStrep tag (IBA Lifesciences, 2–1507-001), Goat Alexa Fluor Plus 488 anti mouse (Thermo Fisher Scientific, A32723), and Goat Alexa Fluor Plus 488 anti rabbit (Thermo Fisher Scientific, A32731). sAB-K29 was used to identify K29-linked ubiquitin chains and was purified as previously reported ^77^. All antibody dilutions were determined experimentally.

#### siRNAs

The following siRNA reagents were used: ON-TARGETplus mouse Ccnf siRNA#1 (CCGCAGAGCUAUCGAAUCA), ON-TARGETplus mouse Ccnf siRNA#3 (CUACCGUGGUUGACUAUAA), ON-TARGETplus mouse Keap1 siRNA (GCGCCAAUGUUGACACGGA), ON-TARGETplus mouse Trip12 siRNA#2 (CGGCAGAGAGAUCCGGUUA), ON-TARGETplus mouse Trip12 siRNA#4 (CGCCUAGAU UGGAUAGAAA), ON-TARGETplus mouse Sqstm1 siRNA (GAACAGAUGGAGUCGGGAA), ON-TARGETplus mouse Fbxo28 siRNA (GCACAUUACAUACGGAUUU), ON-TARGETplus mouse Lrwd1 siRNA GACAAAGAGUCGAUGGGCU), ON-TARGETplus mouse Nfe2l2 siRNA (CAUGUUACGUGAUGAGGAU), ON-TARGETplus Non-targeting Control siRNA#3 (UGGUUUACAUGUUUUCUGA), ON-TARGETplus mouse Ubr5 siRNA SMARTPool (GUAUGAGAGUUUACGACAA, GUUCUUGACAUUCGGAUUU, ACUUGUAU UUCUCGACUUU, CGGUGGUACCUUAAAGAGA).

#### Recombinant proteins

Human NRF2, KEAP1, and p62/SQSTM1 were cloned into pET28a with N-terminal 6xHIS tags (for p62/SQSTM1 6xHIS-MBP) followed by a TEV protease site to allow for tag removal. HIS-tagged proteins were purified from *E. coli* LOBSTR cells grown to OD 0.6 at 37°C and induced overnight at 16 C with 500μM IPTG. Cells were lysed in 50mM HEPES, pH7.5, 150mM NaCl, 1mM PMSF, 5mM β-mercaptoethanol, 10mg/ml lysozyme, 10mM imidazole) for 30min at 4°C, sonicated, and cleared by centrifuging at 20000g for 45min at 4°C. Cleared lysate was mixed with Ni-NTA slurry and incubated at 4°C for 2h. Ni-NTA resin was washed in 50mM HEPES, pH7.5, 150mM NaCl, 5mM β-mercaptoethanol, 20mM imidazole, three times for 15min each at 4°C. Proteins were eluted in 50mM HEPES, pH7.5, 150mM NaCl, 5mM β-mercaptoethanol, 250mM imidazole, and mixed with TEV protease overnight to remove tags. Proteins were dialyzed overnight into 50mM HEPES 7.5, 150 mM NaCl, 5 mM β-mercaptoethanol and re-incubated with Ni-NTA resin to remove tags and uncleaved proteins. Next, proteins were concentrated, analyzed for purity via Coomassie staining, and flash frozen for later use. Recombinant p62/SQSTM1 was never frozen and used for *in vitro* ubiquitylation assays immediately after purification and cleaving off the HIS-MBP tags. CUL3 and RBX1 were co-purified out of SF9 insect cells via strep pull down using a 2xStrep tag on CUL3. Strep pull downs are described above. RBX1 was untagged. After binding CUL3/RBX1 to strep resin and washing, CUL3/RBX1 was eluted using Buffer BXT Strep Elution Buffer (IBA Lifesciences, 2–1042-025), concentrated, analyzed for purity via Coomassie staining, and flash frozen for later use. The antibody recognizing K29 ubiquitin linkages (sAB-K29) was purified as described ^77^. E1 (UBA1), E2 (UBE2D3), E2 (UBE2L3), and neddylation machinery (UBA3/APPBP1, NEDD8, UBE2M) were purified as described ^96,101,102^. Ubiquitin and ubiquitin mutants were purchased from R&D Systems.

#### *In vitro* ubiquitylation

For *in vitro* ubiquitylations using TRIP12, pLVX-2xStrep-TRIP12 plasmids were transfected into three 15cm plates per condition and purified using Strep affinity-agarose as described above. For these reactions, all other components were added directly to strep resin that had bound TRIP12. All other *in vitro* ubiquitylations were performed in solution. Ubiquitylation assays were performed in a 10μl reaction volume with the following protein and buffer conditions: 1μM E1/UBA1, 1μM E2/UBE2D3, 1mg/ml ubiquitin (R&D Systems, U-100H), 10mM DTT, 1x energy mix (22.5mM creatine phosphate (Sigma-Aldrich, 10621714001– 5G), 3mM ATP, 3mM MgCl_2_, 0.3mM EGTA, pH 7.5), 1× ubiquitylation assay buffer (25mM Tris, pH 7.5, 50mM NaCl, 10mM MgCl_2_), and 1μM substrate. PBS was used to fill to 10μl total volume. If CUL3^KEAP1^ were used in the reaction, 1μM NEDD8-modified CUL3/RBX1 and 1μM KEAP1 were added. CUL3/RBX1 were modified with NEDD8 by incubating in 25mM Tris, pH 7.5, 50mM NaCl, 10mM MgCl2, 1x energy mix (22.5mM creatine phosphate (Sigma-Aldrich, 10621714001– 5G), 3mM ATP, 3mM MgCl_2_, 0.3mM EGTA, pH 7.5), 25μM Nedd8, 500μM DTT, 7μM CUL3 complexes, 500nM UBA3/APPBP1, 1μM UBE2M for 30min at 30°C. If p62 was used as a substrate, the MBP tag was cleaved overnight using TEV protease and TEV and uncleaved ^HIS-MBP^p62 were nickel subtracted away leaving only full-length p62/SQSTM1. After adding all components, *in vitro* ubiquitylations were incubated for 2h at 30°C with shaking, before the reaction was stopped by adding 2x urea sample buffer. Samples were analyzed by SDS-PAGE and Western. Ubiquitin linkage specificity was tested using commercially available recombinant human ubiquitin mutants (R&D Systems, UM-K6R, UM-K11R, UM-K27R, UM-K29R, UM-K33R, UM-K48R, UM-K63R, UM- NOK, UM-K60, UM-K110, UM-K270, UM-K290, UM-K330, UM-K480, and UM-K630).

For ^HIS^NRF2 purification after *in vitro* ubiquitylation, NRF2 was purified as described, but the HIS-tag was not removed after purification. All other enzymes used in this reaction had HIS or other purification tags removed to prevent isolation of unwanted proteins. After ubiquitylation, 5% of the reaction was saved as input, and the rest was diluted into 500μl 50mM HEPES, pH 7.5, 150mM NaCl, 5mM β-mercaptoethanol, and incubated with Ni-NTA resin for 1h at 4°C. After incubation, resin was washed three times in buffer, resuspended in 2x urea sample buffer and analyzed via Western blotting.

#### *In vitro* binding assays

TRIP12 was affinity purified and immobilized on strep resin as described above. For control samples, strep resin incubated in cell lysate from mock transfected cells was used. The following recombinant proteins were purified as described above and incubated with either TRIP12 or control resin at concentrations of 500 nM: KEAP1, CUL3, NRF2, Ub-NRF2. The buffer used for these experiments was the same cell lysis buffer used for all pull-down experiments (20 mM HEPES pH 7.5, 150 mM NaCl, 0.2% Nonidet P-40 (Sigma-Aldrich, E1014, 1x complete protease inhibitor cocktail (Roche, 11836170001)). Proteins were incubated with resin at 4°C for 30 minutes and washed five times in lysis buffer. Samples were analyzed by western blotting.

#### siRNA screens

C2C12 myoblasts were seeded into 96-well plates with 400 cells per well using a Thermo Fisher Scientific Multidrop Combi system. 24h later, cells were transfected with 30nM siRNA using an Agilent Velocity 11 Bravo Automated Liquid Handling Platform. 24h later, and each day for 4d, cells were differentiated by changing media once daily into fresh differentiation media. Then, cells were fixed in-well with 4% formaldehyde in 1x PBS for 20min, permeabilized with 0.1% triton in 1xPBS for 20min, blocked in 10% FBS in 1xPBS for 30min, and stained with an antibody recognizing myosin heavy chain and Hoescht33342. Myotubes were imaged on an Opera Phenix (PerkinElmer) using a 10x objective to capture 25 images per well. Images were analyzed using the PerkinElmer Harmony software to calculate the fusion index as previously described ^34^.

#### Myogenesis functional assays

C2C12 myoblasts were grown to 70–90% percent confluence in a 12-well plate, transfected with siRNAs, and media changed into differentiation media as described above. After 3–4d, cells were fixed in-well with 4% formaldehyde in 1x PBS for 20min, permeabilized with 0.1% triton in 1x PBS for 20min, blocked in 10% FBS in 1x PBS for 30min, and stained with primary antibody in 10% FBS and 1x PBS for 3h. After one wash in 1x PBS, secondary antibody and Hoescht33342 in 10% FBS and 1x PBS were added to cells for 1h. All steps were done at room temperature. Cells were imaged in-well using a Perkin Elmer Opera Phenix and images were analyzed using Harmony image analysis software.

#### Immunofluorescence microscopy

C2C12 cells were seeded at 5000 cells/ml on coverslips and transfected with siRNAs as described above. Cells were fixed with 4% formaldehyde in 1x PBS for 20min, permeabilized with 0.1% triton in 1x PBS for 20min, blocked in 10% FBS in 1xPBS for 30min, and stained with primary antibody in 10% FBS and 1x PBS for 3h. After one wash in 1x PBS, secondary antibody and Hoescht33342 in 10% FBS and 1xPBS were added to cells for 1h. All steps were done at room temperature. Coverslips were then mounted onto slides with Prolong Gold Antifade Reagent (Thermo Fisher Scientific, P36930) and imaged on an Olympus IX81 microscope equipped with a Yokogawa CSU-1X confocal scanner unit (CSUX1 Borealis Square Upgrade Module), ANDOR iXon3 camera (IXON DU-897-BV), and Andor Technology Laser Combiner System 500 series equipped with four laser lines. Images were analyzed in FIJI ^97^. For NRF2 and TRIP12 nuclear localization, a mask was created using the Hoescht33342 stain to identify the nucleus and the average NRF2 or TRIP12 signal intensity in the masked area was quantified. NRF2 and TRIP12 nuclear localization experiments consist of at least 100 cells from two biological replicates. For TRIP12 microscopy, 15 uM BSO or 25 uM arsenite were added for 16 hours. For p62 aggregation, the intensity of the p62 aggregates was multiplied by the area of the aggregates and then normalized to the number of cells in the frame. p62 aggregation experiments consist of three biological replicates.

#### RNA sequencing

C2C12 cells were transfected with siRNAs as described above and RNA of three biological replicates was extracted using a NucleosSpin RNA kit (Machery-Nagel, 740955). Library prep, sequencing, and analysis were performed by Novogene. Genes showing a greater than two-fold change compared to siCNTRL were kept for further analysis. Hierarchical clustering was performed and visualized by Morpheus (Morpheus, https://software.broadinstitute.org/morpheus). NRF2 targets ^103^ and E2F targets ^104^ were mapped onto the final heatmap after clustering.

#### DepMap

The Pearson correlations for all genes and either KEAP1 or CUL3 were downloaded from DepMap (https://depmap.org/portal).^66^ Correlations were calculated using the DepMap Public 24Q2+Score, Chronos dataset. The complete list of correlations was compared to a list of E3 ubiquitin ligases^105^ to isolate ligases that may be related to KEAP1 and CUL3. Only positive correlations are shown.

#### ROS measurements

Cellular levels of H_2_O_2_ were determined using the ROS-Glo^™^ H_2_O_2_ Assay (Promega, G8820) according to manufacturer’s instructions.

#### qPCR

C2C12 cells were transfected with siRNAs and RNA was extracted as described above. cDNA was synthesized using Protoscript II First Strand cDNA Synthesis Kit (NEB, E6560S). qPCR assays were done using 2xKAPA SYBR Fast qPCR master mix (Roche, KK4602) on a LightCycler 480 II Instrument (Roche). Expression fold changes were calculated using the ΔΔCt method.

#### Cell competition assays

C2C12 myoblasts were transduced to express either GFP or mCherry by infecting with pLVX-GFP-P2A-Blast or pLVX-mCherry-P2A-Blast viruses as described above. GFP-expressing cells were transfected with siCNTRL and mCherry-expressing cells were transfected with other siRNAs as described in Figures. 12–16h after siRNA transfection, GFP- and mCherry-expressing cells were counted and seeded with 50000 cells per well in a 12-well plate. 8h after seeding, drugs were added to each well. The ratio of GFP^+^/mCherry^+^ cells was determined 48h later using a BD LSRFortessa instrument, analyzed using FlowJo, and normalized to untreated siCNTRL sample. The ratio is calculated as ((siRNA_treatment_/siCONTROL_treatment_)/(siRNA_control_/siCONTROL_control_)) where siRNA_treatment_ is any non-siCNTRL sample.

### QUANTIFICATION AND STATISTICAL ANALYSIS

The quantifications presented in this study are shown as the mean ± standard deviation (SD). Myogenesis screens (Figures 1A and 2A) are two technical replicates. All other myogenesis experiments are three biological replicates. RNA-seq analysis was done using three biological replicates. All qPCR is three technical replicates. NRF2 and TRIP12 microscopy are at least 100 cells from two biological replicates and p62 microscopy is three biological replicates. All myogenesis experiments, immunofluorescence microscopy, and qPCR were analyzed for significance in GraphPad Prism by one-way ANOVA to compare all conditions at once (* p ≤ 0.05, ** p ≤ 0.01, *** p ≤ 0.001, **** p ≤ 0.0001).

## Supplementary Material

1

2

3

SUPPLEMENTAL INFORMATION

Supplemental information can be found online at https://doi.org/10.1016/j.celrep.2025.116262.

## Figures and Tables

**KEY RESOURCES TABLE:** 

REAGENT or RESOURCE	SOURCE	IDENTIFIER
Antibodies		
Rabbit monoclonal anti-NRF2	Cell Signaling	Cat# 12721S; RRID: AB_2715528
Rabbit monoclonal anti-KEAP1	Cell Signaling	Cat# 7705S; RRID: AB_10860422
Rabbit polyclonal anti-TRIP12	Proteintech	Cat# 25303-1-AP; RRID: AB_2880020
Mouse monoclonal anti-SQSTM1	Abcam	Cat# ab56416; RRID: AB_945626
Rabbit monoclonal anti-GAPDH	Cell Signaling	Cat# 14C10; RRID: AB_561053
Mouse monoclonal anti-beta-actin	MP Biomedicals	Cat# 08691001; Clone C4; RRID: AB_2335304
Mouse monoclonal anti-myosin heavy chain, sarcomere (MHC)	Developmental Studies Hybridoma Bank (DHSB)	Clone MF20; RRID: AB_1293549
Mouse monoclonal anti-CUL3 generated with Covance	Mena et al.^96^	N/A
Mouse monoclonal anti-Strep tag	IBA Lifesciences	Cat# 2-1507-001; RRID: AB_513133
Goat Alexa Fluor Plus 488 anti mouse	Thermo Fisher Scientific	Cat# A32723
Goat Alexa Fluor Plus 488 anti rabbit	Thermo Fisher Scientific	Cat# A32731
Bacterial and virus strains		
E.coli: One Shot Stbl3 Chemically competent cells	Thermo Fisher Scientific	Cat# C7373-03
E. coli: DH5alpha	Thermo Fisher Scientific	Cat# 18265017
E. coli LOBSTR Cells	Kerafast	Cat# EC1002
Chemicals, peptides, and recombinant proteins		
KEAP1	This paper	N/A
NRF2	This paper	N/A
UbiquitinΔgg-NRF2	This paper	N/A
Ubiquitin(K29R)Δgg-NRF2	This paper	N/A
MBP-p62/SQSTM1	This paper	N/A
CUL3/RBX1	This paper	N/A
sAB-K29	Yu et al.^77^	RRID: addgene_204735
E1/UBA1	Laboratory of Michael Rape	N/A
E2/UBE2D3	Laboratory of Michael Rape	N/A
E2/UBE2L3	Laboratory of Michael Rape	N/A
NEDD8	Laboratory of Michael Rape	N/A
UBA3/APPBP1	Laboratory of Michael Rape	N/A
UBE2M	Laboratory of Michael Rape	N/A
TEV protease	UC Berkeley QB3 MacroLab	N/A
Carfilzomib	Selleck Chemical	Cat# PR-171
TCEP (Tris(2-carboxyethyl)phosphine hydrochloride))	Sigma-Aldrich	Cat# C4706
Iodoacetamide	Thermo Fisher Scientific	Cat# A39271
3xFlag peptide	Millipore	Cat# F4799
Phenylmethanesulfonyl fluoride	Sigma-Aldrich	Cat# P7626
Pevonedistat (MLN4924)	Selleck Chemical	Cat# S7109
Alpha-keto glutatarate	Sigma-Aldrich	Cat# 349631-5G
Succinate	Sigma-Aldrich	Cat# 112402-250G
Oligomycin A	Santa Cruz Biotechnology	Cat# sc-201551
Antimycin A	Santa Cruz Biotechnology	Cat# sc-202467
DL-Buthionine-(S,R)-sulfoximine (BSO)	Millipore Sigma	Cat# B2640-500MG
Auranofin	Selleck Chemicals	Cat# S4307
Deferiprone (DFP)	Selleck Chemicals	Cat# S4067
Hydrogen peroxide	Fisher chemical	Cat# H325-500
Sodium arsenite	Ricca Chemical	Cat# 714216
Piericidin A	Cayman Chemical	Cat# 15379
Rotenone	Sigma-Aldrich	Cat# R8875-1G
Mitoparaquat	Selleck Chemicals	Cat# E1252
TBHQ	Selleck Chemicals	Cat# S4990
Bardoxolone Methyl	Selleck Chemicals	Cat# S8078
CHIR-98014	Selleck Chemicals	Cat# S2745
Bafilomycin A1	Selleck Chemicals	Cat# S1413
Critical commercial assays		
Pierce 660nm Protein Assay Reagent	Thermo Fisher Scientific	Cat# 22660
Ionic Detergent Compatibility Reagent	Thermo Fisher Scientific	Cat# 22663
ROS-Glo^™^ H2O2 Assay	Promega	Cat# G8820
Deposited data		
RNA-seq of C2C12 myoblasts depleted of either KEAP1, CCNF, or both	GSE281184O	N/A
Proteomic data; MassIVE	MSV000098529	N/A
Experimental models: Cell lines		
C2C12	ATCC	Cat# CRL-1772; RRID:CVCL_0188
HEK293T	ATCC	Cat# CRL-3216; RRID: CVCL_0063
Oligonucleotides		
ON-TARGETplus Mouse Ccnf siRNA #1	Horizon Discovery	Cat# J-060284-05
ON-TARGETplus Mouse Ccnf siRNA #3	Horizon Discovery	Cat# J-060284-07
ON-TARGETplus Mouse Keap1 siRNA	Horizon Discovery	Cat# J-041104-09
ON-TARGETplus Mouse Trip12 siRNA #2	Horizon Discovery	Cat# J-053913-10
ON-TARGETplus Mouse Trip12 siRNA #4	Horizon Discovery	Cat# J-053913-12
ON-TARGETplus Mouse Sqstm1 siRNA	Horizon Discovery	Cat# J-047628-10
ON-TARGETplus Mouse Fbxo28 siRNA	Horizon Discovery	Cat# J-059143-11
ON-TARGETplus Mouse Lrwd1 siRNA	Horizon Discovery	Cat# J-047012-11
ON-TARGETplus Mouse Nfe2l2 siRNA	Horizon Discovery	Cat# J-040766-07
ON-TARGETplus Mouse Ubr5 siRNA SMARTPool	Horizon Discovery	Cat# L-048858-01
ON-TARGETplus Mouse Fbxw11 siRNA SMARTPool	Horizon Discovery	Cat# L-058886-00
ON-TARGETplus Non-targeting Control siRNA #3	Horizon Discovery	Cat# D-001810-03
Recombinant DNA		
pCS2-KEAP1-FLAG	This paper	N/A
pLVX-FLAG-NRF2-IRES-Puro	This paper	N/A
pLVX-FLAG-NRF2-ETGE/AAAA	This paper	N/A
pLVX-2xStrep-TRIP12-IRES-Puro	This paper	N/A
pLVX-2xSTREP-TRIP12ΔIDR-IRES-Puro	This paper	N/A
pLVX-2xStrep-TRIP12ΔIDR-C/S-IRES-Puro	This paper	N/A
pLVX-2xStrep-CUL3-IRES-Puro	This paper	N/A
pLVX-GFP-P2A-BLAST	Haakonsen et al.^55^	N/A
pLVX-mCherry-P2A-BLAST	Haakonsen et al.^55^	N/A
pET28a-HIS-TEV-KEAP 1	This paper	N/A
pET28a-HIS-TEV-NRF2	This paper	N/A
pET28a-HIS-Thr-UBΔgg-NRF2	This paper	N/A
pET28a-HIS-Thr-UB(K29R)Δgg-NRF2	This paper	N/A
pET28a-HIS-Thr-MBP-TEV-p62/SQSTM1	This paper	N/A
pFastBac-2xStrep-TEV-HA-CUL3	This paper	N/A
pFastBac-RBX1	Laboratory of Michael Rape	N/A
sAB-K29	Yu et al.^77^	RRID: addgene_204735
Software and algorithms		
Metamorph Advanced	Molecular Devices	RRID:SCR_002368
Harmony High-Content Imaging and Analysis Software	PerkinElmer	Cat# HH17000010
FIJI	Schindelin et al.^97^	RRID:SCR_002285
CompPASS	Huttlin et al.^98^	N/A
FlowJo	BD	RRID:SCR_008520
Prism	GraphPad	RRID:SCR_002798
Other		
Lipofectamine RNAiMAX	ThermoFisher Scientific	Cat# 13778030
Lipofectamine 3000	ThermoFisher Scientific	Cat# L3000008
Complete, EDTA-free protease inhibitor cocktail tablets from Roche	Sigma-Aldrich	Cat# 11873580001
Hoechst 33342	AnaSpec	Cat# 83218
Opti-MEM (1X) Reduced Serum Medium	GIBCO	Cat# 31985-070
DMEM (1X) +GlutaMAXTM-I	GIBCO	Cat# 51985091
Trypsin-EDTA (0.25%)	GIBCO	Cat# 25200056
Fetal Bovine Serum	VWR LifeScience	Cat# 97068-107
Ni-NTA	QIAGEN	Cat# 30210
ANTI-FLAG M2 Affinity Agarose Gel slurry	Sigma-Aldrich	Cat# CA2220
Strep-Tactin^®^XT 4Flow^®^ resin	IBA Lifesciences	Cat# 2-5010-025
Buffer BXT (10x)	IBA Lifesciences GmbH	Cat# 2-1042-025
Protein G-Agarose	Sigma-Aldrich	Cat# 11243233001
Lenti-X concentrator	Takara	Cat# 631232
PrimeSTAR GXL DNA Polymerase	Takara	Cat# R050A
Prolong Gold Antifade Reagent	Thermo Fisher Scientific	Cat# P36930

## Highlights

- TRIP12 binds CUL3^KEAP1^
- TRIP12 elongates ubiquitin chains on NRF2
- TRIP12 ensures robust NRF2 degradation and stress response silencing
- TRIP12 inhibition boosts cell survival during oxidative stress
