# Supplementary material for: Dynamic regulation of the oxidative stress response by the E3 ligase TRIP12
Source: Cell Rep. Author manuscript; Available in PMC 2025 Oct 28. (PMC12560144; doi:10.1016/j.celrep.2025.116262)
Supplement: 1 [file NIHMS2113409-supplement-1.pdf]

Cell Reports, Volume *44*

Supplemental information

Dynamic regulation of the oxidative stress response by the E3 ligase TRIP12

Andrew J. Ingersoll, Devlon M. McCloud, Jenny Y. Hu, and Michael Rape

Supplementary Figures and Legends:

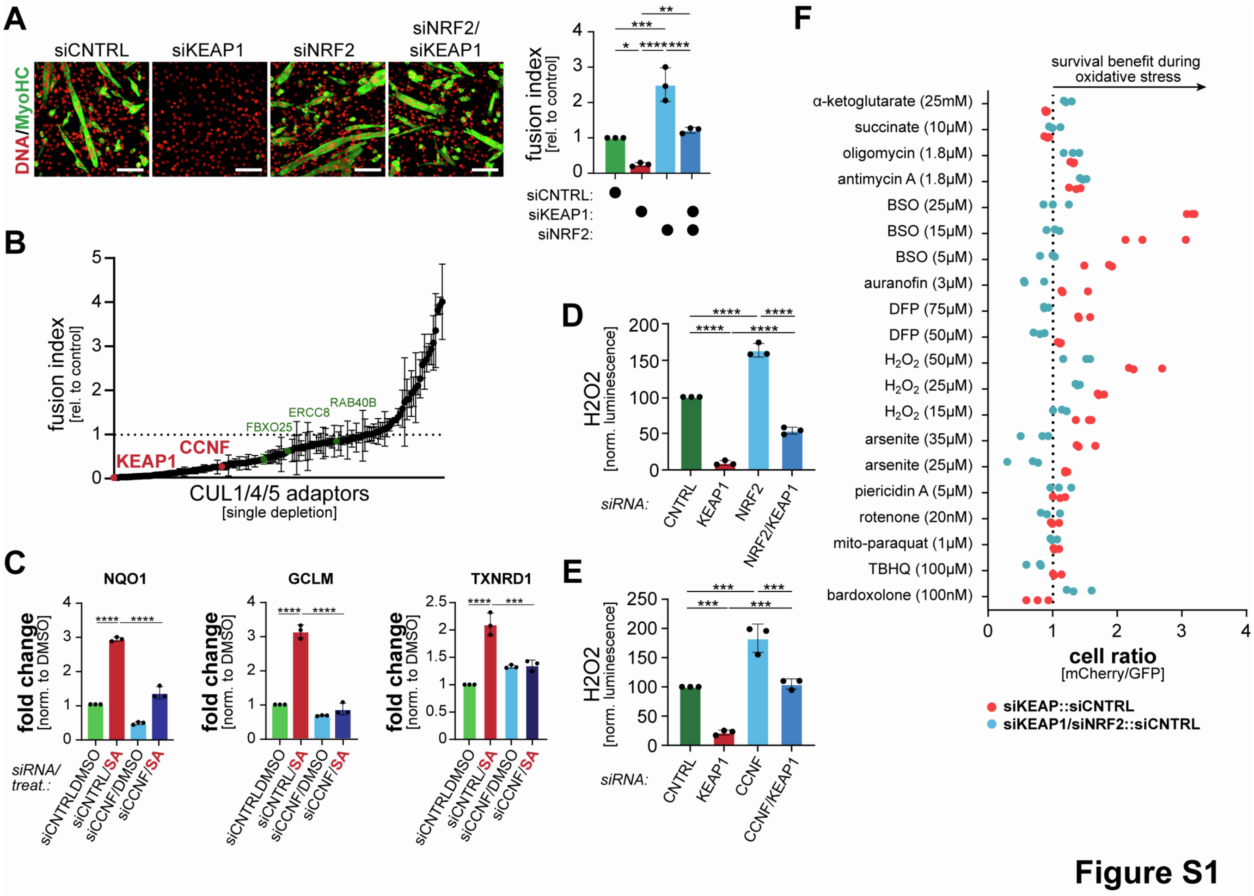

Figure S1, related to Figure 1: CCNF modulates oxidative stress signaling in myoblasts. A. C2C12 myoblasts were depleted of KEAP1, NRF2, or both. After differentiation, myotube formation was analyzed by immunofluorescence against MyoHC (green). Nuclei were stained with Hoechst (red). Quantification of three independent experiments is shown on the right. Data is represented as mean ± standard deviation. * $p<0.05$; ** $p<0.01$; *** $p<0.001$; **** $p<0.0001$. **B.** Substrate adaptors of CUL1, CUL4, and CUL5 E3 ligases were depleted from C2C12 myoblasts. After differentiation was initiated, the success of myotube formation was monitored by immunofluorescence microscopy against MyoHC. n=2 replicates. **C.** C2C12 myoblasts were

transfected with control siRNAs or siRNAs against CCNF and exposed to sodium arsenite (16h; 25µM). Expression of select NRF2 targets was determined by qPCR. n=3 replicates. Data is represented as mean ± standard deviation. *** $p<0.001$; **** $p<0.0001$. **D.** C2C12 myoblasts were depleted of KEAP1, NRF2, or both, and intracellular ROS were determined by a ROS-Glo™ H_2O_2 assay (Promega). Data is represented as mean ± standard deviation. **** $p<0.0001$. **E.** C2C12 myoblasts were depleted of KEAP1, CCNF, or both, and intracellular ROS were determined by a ROS-Glo™ H_2O_2 assay (Promega). Data is represented as mean ± standard deviation. *** $p<0.001$ **F.** GFP-labeled control cells were mixed at a 1:1 ratio with mCherry-labeled cells depleted of KEAP1 (red dots). As indicated, NRF2 was also depleted (blue dots). Cells were exposed to increasing concentrations of oxidative stressors. After two days, the ratio of GFP- to mCherry-labeled cells was determined by flow cytometry. n=3 independent experiments. KEAP1-depleted cells are the same as shown in Figure 1F.

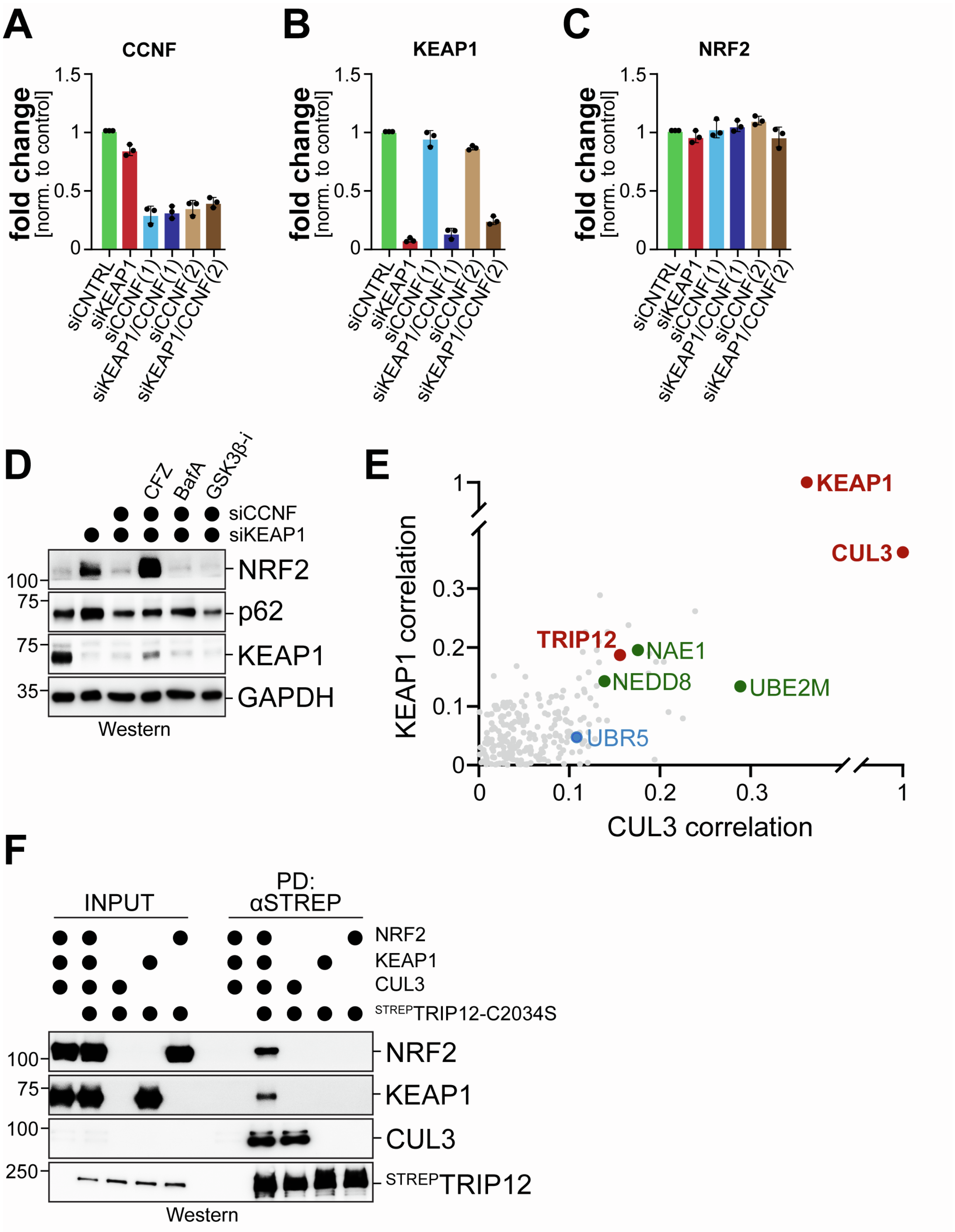
A
CCNF
B
KEAP1
C
NRF2
fold change
[norm. to control]
1.5
1
0.5
0
siCNTRL
siKEAP1
siCCNF(1)
siKEAP1/CCNF(1)
siCCNF(2)
siKEAP1/CCNF(2)
D
CFZ
BafA
GSK3β-i
siCCNF
siKEAP1
NRF2
p62
KEAP1
GAPDH
100
75
75
35
Western
E
KEAP1
CUL3
TRIP12
NAE1
NEDD8
UBE2M
UBR5
KEAP1 correlation
CUL3 correlation
0
0.1
0.2
0.3
1
F
INPUT
PD:
αSTREP
NRF2
KEAP1
CUL3
STREPTRIP12-C2034S
NRF2
KEAP1
CUL3
STREPTRIP12
100
75
100
250
Western

Figure S2

Figure S2, related to Figure 3: Depletion of CCNF does not affect NRF2 mRNA. A. C2C12 myoblasts were depleted of KEAP1, CCNF (2 independent siRNAs), or both, and the abundance of CCNF mRNA was determined by qPCR. Data is represented as mean ± standard deviation. **B.** C2C12 myoblasts were depleted of KEAP1, CCNF (2 independent siRNAs), or both, and the abundance of KEAP1 mRNA was determined by qPCR. Data is represented as mean ± standard deviation. **C.** C2C12 myoblasts were depleted of KEAP1, CCNF (2 independent siRNAs), or both, and the abundance of NRF2 mRNA was determined by qPCR. Data is represented as mean ± standard deviation. **D.** C2C12 myoblasts were depleted of KEAP1 or KEAP1 and CCNF at the same time and treated with the proteasome inhibitor carfil-zomib; the lysosome inhibitor bafilomycin A (BafA) or the GSK3β-inhibitor CHIR98014. NRF2 levels were determined by Western blotting. **E.** DepMap analyses reveal TRIP12 as one of the most strongly correlated E3 ligases with CUL3 and KEAP1. Shown are all positive correlations of E3 ligases with either KEAP1 or CUL3, as derived from DepMap. **F.** Catalytically inactive TRIP12^{C2034S} directly binds CUL3. Immobilized ^{STREP}TRIP12^{C2034S} was incubated with recombinant CUL3, KEAP1, NRF2, or all proteins at the same time, and bound proteins were detected by Western blotting.

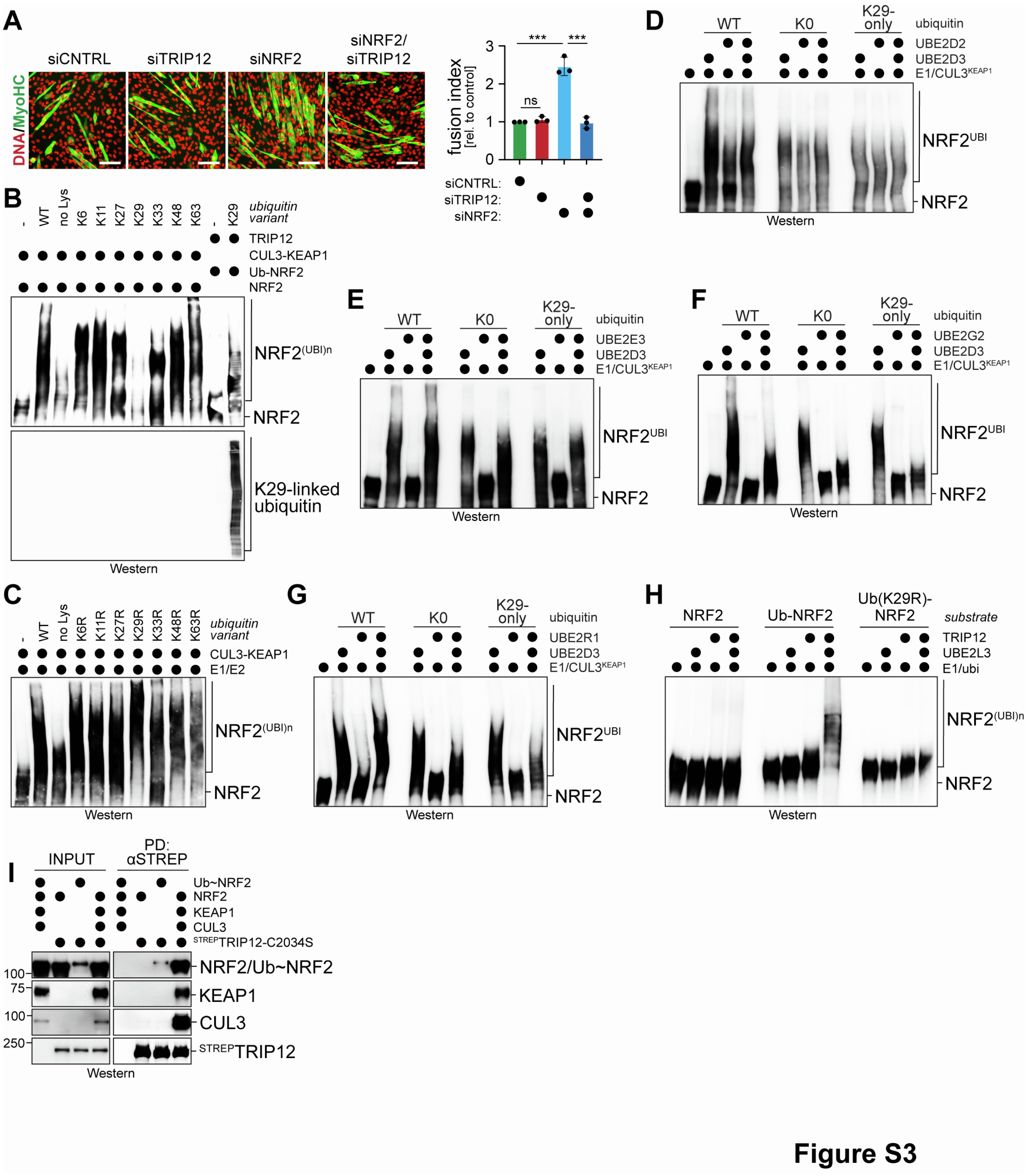

Figure S3, related to Figure 4: TRIP12 is a ubiquitin chain elongation factor for NRF2. A. C2C12 myoblasts were depleted of NRF2, TRIP12, or both. Following differentiation, the success of myotube formation was monitored by immunofluorescence against MyoHC. Quantification of three independent experiments is shown on the right. **B.** Data is represented as mean ± standard

deviation. *** $p<0.001$ CUL3^{KEAP1} does not assemble ubiquitin conjugates containing K29-linkages. Purified NRF2 was incubated with CUL3^{KEAP1}, E1, UBE2D3, and either wildtype or single-Lys ubiquitin mutants. In the last two lanes, purified Ub~NRF2 was incubated with TRIP12 and a ubiquitin variant containing Lys29 (positive control for K29-linkage formation). Reaction products were analyzed by Western blotting using antibodies against NRF2 or K29-linked ubiquitin chains. **C.** CUL3^{KEAP1}-dependent ubiquitylation of NRF2 occurs more efficiently in the presence of a ubiquitin variant lacking K29 (ubi-K29R). Ubiquitylation of purified NRF2 was performed as described above. **D.** Ubiquitylation of NRF2 by CUL3^{KEAP1} in the presence of UBE2D2, UBE2D3, or both E2 enzymes, as well as wt-ubiquitin, ubiquitin-K0 (all Lys residues mutated to Arg), or ubiquitin-K29only (all Lys residues, except for K29, mutated to Arg). Reaction products were analyzed by Western blotting using αNRF2-antibodies. **E.** Ubiquitylation of NRF2 by CUL3^{KEAP1} in the presence of UBE2E3, UBE2D3, or both E2 enzymes, as well as wt-ubiquitin, ubiquitin-K0 (all Lys residues mutated to Arg), or ubiquitin-K29only (all Lys residues, except for K29, mutated to Arg). Reaction products were analyzed by Western blotting using αNRF2-antibodies. **F.** Ubiquitylation of NRF2 by CUL3^{KEAP1} in the presence of UBE2G2, UBE2D3, or both E2 enzymes, as well as wt-ubiquitin, ubiquitin-K0 (all Lys residues mutated to Arg), or ubiquitin-K29only (all Lys residues, except for K29, mutated to Arg). Reaction products were analyzed by Western blotting using αNRF2-antibodies. **G.** Ubiquitylation of NRF2 by CUL3^{KEAP1} in the presence of UBE2R1, UBE2D3, or both E2 enzymes, as well as wt-ubiquitin, ubiquitin-K0 (all Lys residues mutated to Arg), or ubiquitin-K29only (all Lys residues, except for K29, mutated to Arg). Reaction products were analyzed by Western blotting using αNRF2-antibodies. **H.** TRIP12$^{\Delta IDR}$ was purified from 293T cells and incubated with E1, UBE2L3 as a HECT-specific E2, ubiquitin, and either recombinant NRF2, Ub~NRF2, or Ub^{K29R}~NRF2. Reaction products were analyzed by Western blotting against NRF2. **I.** TRIP12 can directly bind Ub~NRF2, but not NRF2. Immobilized ^{STREP}TRIP12 was incubated with recombinant CUL3 and either NRF2 or Ub~NRF2 and bound

proteins were detected by Western blotting. As indicated, KEAP1 was included in the binding reaction with NRF2 to provide a positive control for NRF2-recognition.

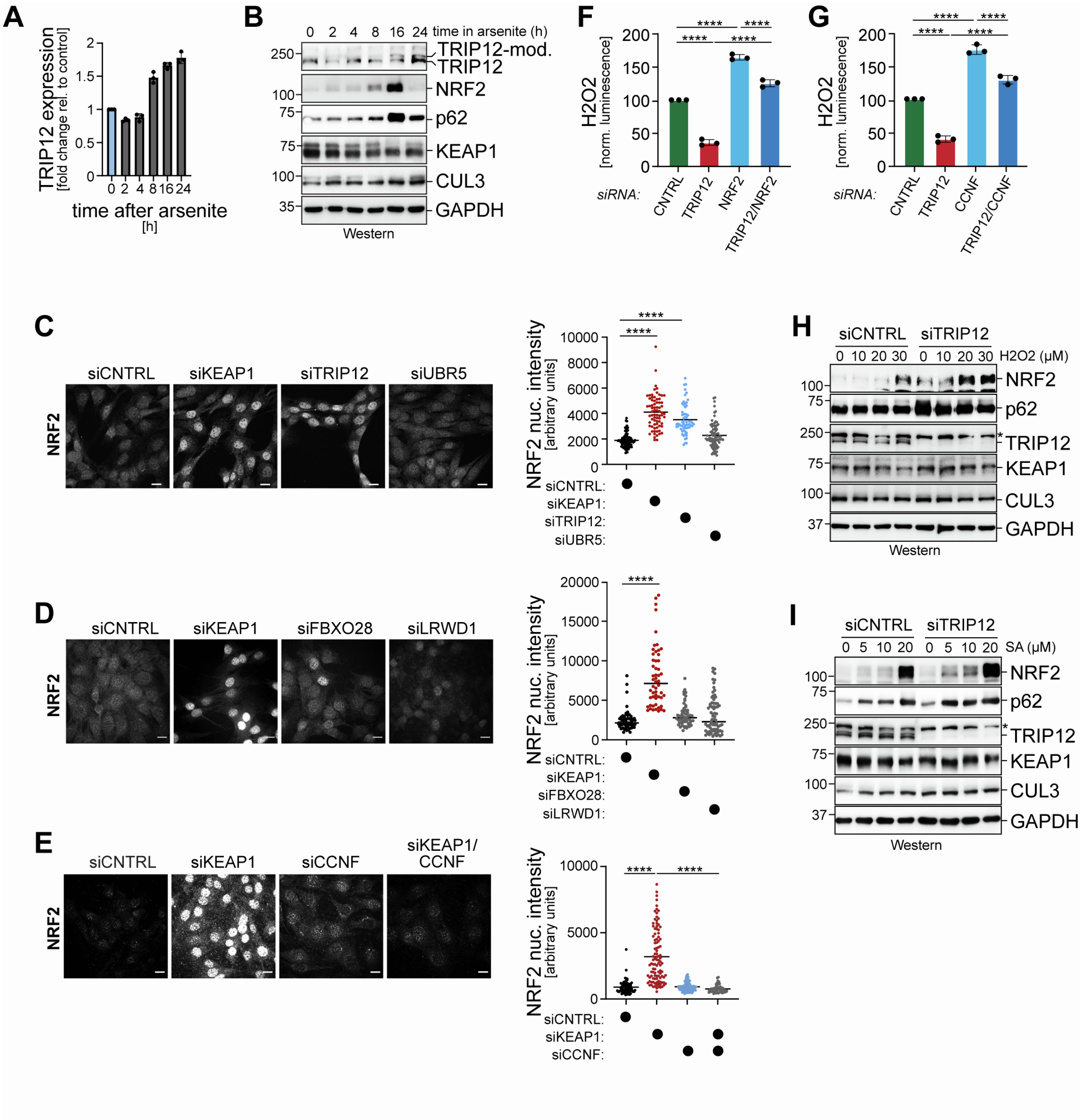

Figure S4

Figure S4, related to Figure 5: TRIP12 restricts NRF2 accumulation. A. qRT-PCR analyses show that TRIP12 increases at times when NRF2 is stabilized during oxidative stress. **B.** TRIP12

protein levels increase late after oxidative stress induction, as shown by Western. **C.** C2C12 myoblasts were depleted of KEAP1, TRIP12, or UBR5, and levels of nuclear NRF2 were determined by immunofluorescence microscopy against endogenous NRF2. Quantification is shown on the right. Data is represented as mean ± standard deviation. **** $p<0.0001$. UBR5 is the E3 ligase that is most closely correlated with TRIP12 across DepMap. **D.** C2C12 myoblasts were depleted of KEAP1, FBXO28, or LRWD1, and levels of nuclear NRF2 were determined by immunofluorescence microscopy against endogenous NRF2. Quantification is shown on the right. Data is represented as mean ± standard deviation. **** $p<0.0001$. FBXO28 and LRWD1 were detected in NRF2 affinity-purification and mass spectrometry. **E.** C2C12 myoblasts were depleted of KEAP1, CCNF, or both, and levels of nuclear NRF2 were determined by immunofluorescence microscopy against endogenous NRF2. Quantification is shown on the right. Data is represented as mean ± standard deviation. **** $p<0.0001$. **F.** C2C12 myoblasts were depleted of TRIP12, NRF2, or both, and intracellular ROS were determined by a ROS-Glo™ H_2O_2 assay (Promega). Data is represented as mean ± standard deviation. **** $p<0.0001$. **G.** C2C12 myoblasts were depleted of TRIP12, CCNF, or both, and intracellular ROS were determined by a ROS-Glo™ H_2O_2 assay (Promega). Data is represented as mean ± standard deviation. **** $p<0.0001$. **H.** C2C12 myoblasts were depleted of TRIP12 and exposed to increasing concentrations of hydrogen peroxide. Levels of NRF2 and additional proteins were determined by Western blotting using specific antibodies. **I.** C2C12 myoblasts were depleted of TRIP12 and exposed to increasing concentrations of sodium arsenite. Levels of NRF2 and additional proteins were determined by Western blotting using specific antibodies.

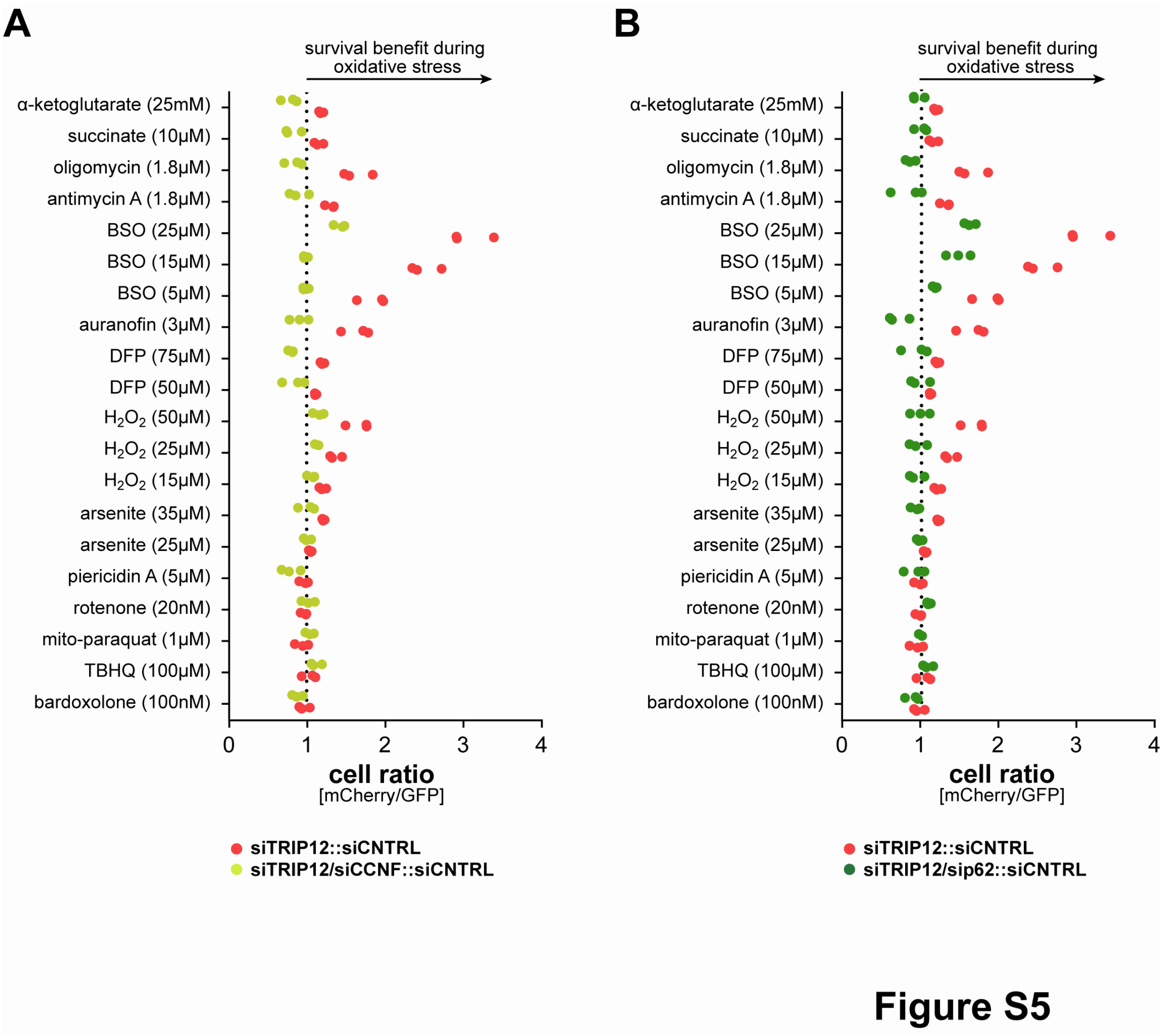

Figure S5, related to Figure 7: TRIP12 depletion promotes cell survival during stress. A. GFP-labeled control cells were mixed at a 1:1 ratio with mCherry-labeled cells depleted of TRIP12 (red dots). As indicated, CCNF was also depleted (yellow dots). Cells were exposed to increasing concentrations of oxidative stressors. After three days, the ratio of GFP- to mCherry-labeled cells was determined by flow cytometry. n=3 independent experiments. TRIP12-depleted cells are the same as shown in Figure 7B. **B.** GFP-labeled control cells were mixed at a 1:1 ratio with mCherry-labeled cells depleted of TRIP12 (red dots). As indicated, p62 was also depleted (green dots). Cells were exposed to increasing concentrations of oxidative stressors. After three days, the ratio

of GFP- to mCherry-labeled cells was determined by flow cytometry. n=3 independent experiments. TRIP12-depleted cells are the same as shown in Figure 7B.

Table S1: siRNA screen to identify regulators of NRF2 stability of function. C2C12 myoblasts were differentiated in the presence of absence of KEAP1 siRNAs. Fusion index was quantified after MyHC immunofluorescence.

Table S2: Analysis of affinity-purification of NRF2 or NRF2$^{\Delta ETGE}$
